# Physiologically based metformin pharmacokinetics model of mice and scale-up to humans for the estimation of concentrations in various tissues

**DOI:** 10.1371/journal.pone.0249594

**Published:** 2021-04-07

**Authors:** Darta Maija Zake, Janis Kurlovics, Linda Zaharenko, Vitalijs Komasilovs, Janis Klovins, Egils Stalidzans

**Affiliations:** 1 Latvian Biomedical Research and Study Centre, Riga, Latvia; 2 Institute of Microbiology and Biotechnology, University of Latvia, Riga, Latvia; 3 Division of Pharmaceutical Chemistry and Technology, University of Helsinki, Helsinki, Finland; University of Colorado Denver Skaggs School of Pharmacy and Pharmaceutical Sciences, UNITED STATES

## Abstract

Metformin is the primary drug for type 2 diabetes treatment and a promising candidate for other disease treatment. It has significant deviations between individuals in therapy efficiency and pharmacokinetics, leading to the administration of an unnecessary overdose or an insufficient dose. There is a lack of data regarding the concentration-time profiles in various human tissues that limits the understanding of pharmacokinetics and hinders the development of precision therapies for individual patients. The physiologically based pharmacokinetic (PBPK) model developed in this study is based on humans’ known physiological parameters (blood flow, tissue volume, and others). The missing tissue-specific pharmacokinetics parameters are estimated by developing a PBPK model of metformin in mice where the concentration time series in various tissues have been measured. Some parameters are adapted from human intestine cell culture experiments. The resulting PBPK model for metformin in humans includes 21 tissues and body fluids compartments and can simulate metformin concentration in the stomach, small intestine, liver, kidney, heart, skeletal muscle adipose, and brain depending on the body weight, dose, and administration regimen. Simulations for humans with a bodyweight of 70kg have been analyzed for doses in the range of 500-1500mg. Most tissues have a half-life (T_1/2_) similar to plasma (3.7h) except for the liver and intestine with shorter T_1/2_ and muscle, kidney, and red blood cells that have longer T_1/2_. The highest maximal concentrations (C_max_) turned out to be in the intestine (absorption process) and kidney (excretion process), followed by the liver. The developed metformin PBPK model for mice does not have a compartment for red blood cells and consists of 20 compartments. The developed human model can be personalized by adapting measurable values (tissue volumes, blood flow) and measuring metformin concentration time-course in blood and urine after a single dose of metformin. The personalized model can be used as a decision support tool for precision therapy development for individuals.

## 1. Introduction

Metformin has been prescribed to treat Type 2 Diabetes mellitus (T2D) since the 1960-s [1]. It is currently the most commonly prescribed drug for T2D, as it is recommended as the first-line medication in nearly all newly diagnosed T2D patients by international treatment guidelines [2, 3]. It is assumed that metformin is used by over 120 million patients worldwide [3–5]. Moreover, recent studies have demonstrated a possible beneficial potential of metformin administration for cancer, obesity, non-alcoholic fatty liver disease, polycystic ovary syndrome, metabolic syndrome patients [6] and could even have neuroprotective effects in Alzheimer’s disease type pathology [7].

Metformin belongs to the peroral (PO) antidiabetic drug class of biguanides. It improves glucose tolerance in patients with T2D, lowering both basal and postprandial plasma glucose levels by reducing hepatic neogenesis in non-insulin-dependent diabetes mellitus patients. Metformin is administered orally as an immediate release or sustained release tablet. It is administered in the form of a hydrochloride salt with an oral bioavailability of 50–60% [8]. Metformin is a hydrophilic base with a high acid dissociation value *(pKa = 11*.*5)*. *It* is present as a cation with less than 0.01% under physiological pH unionized in blood. Despite the high ionization level at a physiological pH and the fact that metformin has a high unbound fraction in plasma [9], after intravenous administration, the volume of distribution (Vd) is around 65 L [10, 11]. It is even higher following a single oral dose where it has been estimated to be above 200 L [9], which suggest that metformin has significant tissue uptake and is expected to rely mainly on transporters like organic cation transporters (OCT) for its movement across cellular membranes [8, 9, 12, 13]. At the same time, metformin can be detected in red blood cells where relevant transporters are not expressed, suggesting a possible transport by diffusion, moreover due to its chemical characteristics, binding with cellular acidic phospholipids could be expected [14, 15]. It is believed that metformin is not metabolized or goes under negligible hepatic metabolism in mice and humans [9, 16]. Moreover, studies investigating the possible formation of metabolites have failed to recognize any potential metabolites of metformin [17].

The doses of metformin hydrochloride used in therapy range from 500 mg up to 3000 mg per day [9], but the effective therapeutic concentrations in metformin action’s significant compartments (such as the intestine, liver, muscle, and adipose tissue) are unknown for human. Thus, adequate therapeutic metformin concentrations for an individual in particular tissues have not been determined or even estimated, leading to an inadequate dose administration with unnecessary stress in the case of over-dosing and limited therapeutic effects in the case of under-dosing. The therapeutic response of metformin varies substantially, with about 30% of patients failing to achieve glycaemic control [13, 18]. While the pharmacokinetics of metformin have been studied in many aspects, the intricacies of the pharmacokinetics are still being elucidated and could be the underlying cause of the varying therapeutic response [8, 11, 12, 15, 17, 19, 20].

Mechanistic mathematical modeling can be applied to parametrize systemic processes under data insufficiency [21]. Physiologically based pharmacokinetic (PBPK) models aim to describe the absorption, distribution, metabolism, and elimination of a drug in a physiologically relevant compartmental structure, where each compartment represents an organ or a tissue. The organs and tissues are connected via arterial and venous blood flow joined in the lungs [22, 23]. The pharmacokinetics of a drug in various organs and tissues are described by ordinary differential equations (ODEs). The plasma-tissue partition coefficients (K_t:p_) are used in PBPK models to describe the distribution of pharmaceutical ingredients across the corresponding tissue compartments [24–26]. It is expected that the K_t:p_ simultaneously represents passive (transport by diffusion) and active (active transporter-mediated transport) components [27]. However, the major limitation of PBPK models is the lack of human data. This limitation could be addressed using *in vivo* data in different species and *in vitro* predictions for both distribution and elimination. One of the most attractive features of the PBPK model is its scalability from one species to another, as it can be assumed that the K_t:p_ are identical across species [14], allowing to use of the estimated K_t:p_ values to extrapolate from laboratory animals to humans. A scale-up strategy used in the current study by extrapolating models of laboratory animals, like mice, rats, dogs, rabbits, and monkeys, is used in several other studies [24–26].

Several PBPK models have been developed to characterize or predict the metformin pharmacokinetics in special populations [28–30] or investigate its transporter-mediated drug–drug interactions and exposure [8, 31–33]. These studies extrapolate the model assumptions from *in vitro* data. In contrast, in our research, we aim to combine *in vitro* data with metformin concentration distribution data in mice tissues from Wilcock and Bailey to develop a whole-body mechanistic PBPK model for humans [1]. There has been no attempt to model metformin distribution by scale-up from an animal model to the best of our knowledge. This study’s focus is on the metformin distribution in the human body–the concentrations reached in the major tissues of metformin action. The developed PBPK model predicts the drug’s tissue distribution and explains some underlying differences in the individual responses to metformin therapy. The developed PBPK models for mice and humans provide a flexible tool for integrating the currently available biological knowledge and testing various hypotheses *in silico*.

## 2. Results

PBPK model for mice has been developed and parametrized using available experimental data to apply the found parameters for the human model. Both models are described in detail separately.

### 2.1. Metformin PBPK model in mice

#### 2.1.1. Parameter estimation

**Fig 1:**
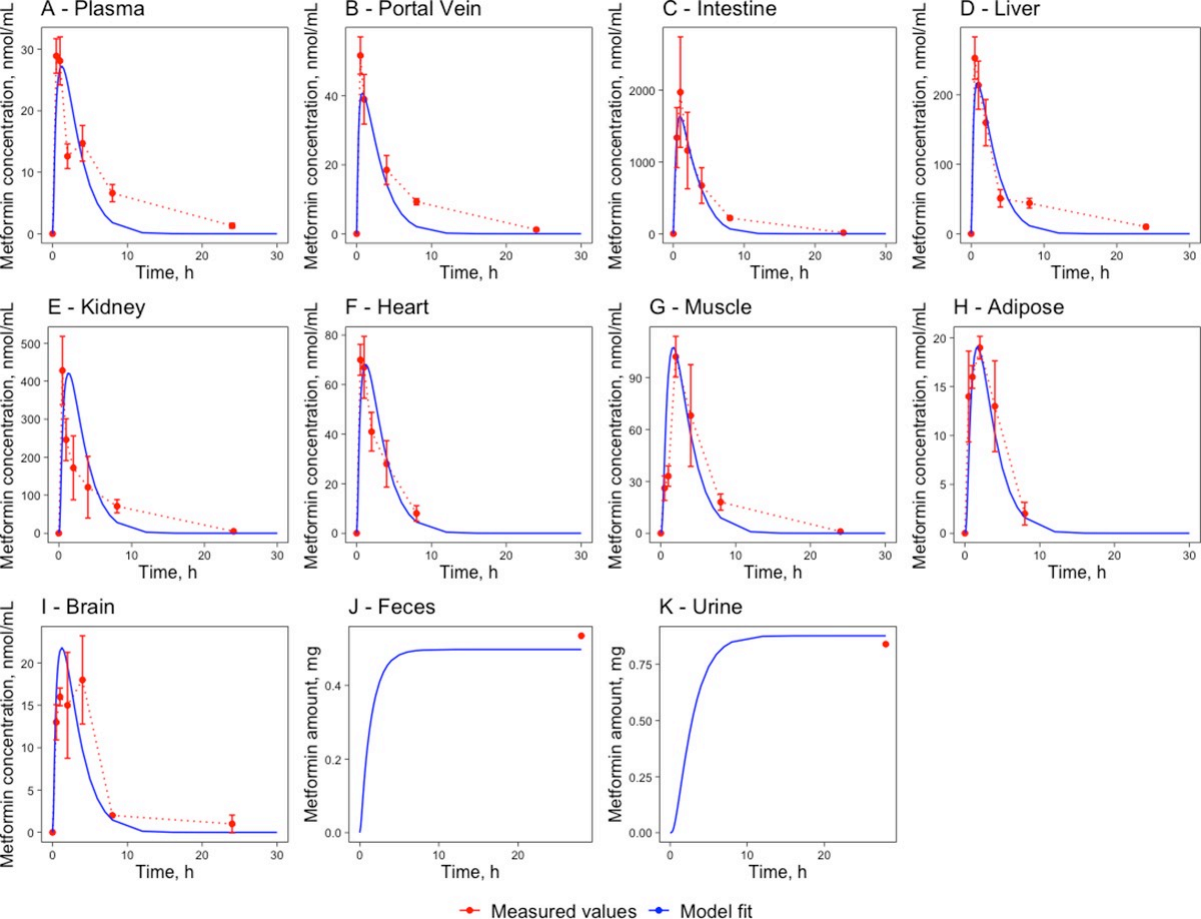
Metformin pharmacokinetics in major compartments of metformin action. Venous plasma (A), portal vein (B), small intestine (C), liver (D), kidney (E), heart (F), muscle (G), adipose (H), brain (I), feces (J) and urine (K) following a single PO 50 mg/kg dose in mice. The red marks represent the experimental data’s concentration-time profiles with error bars representing standard deviation [1] and the blue lines represent the model simulations.

**Fig 2:**
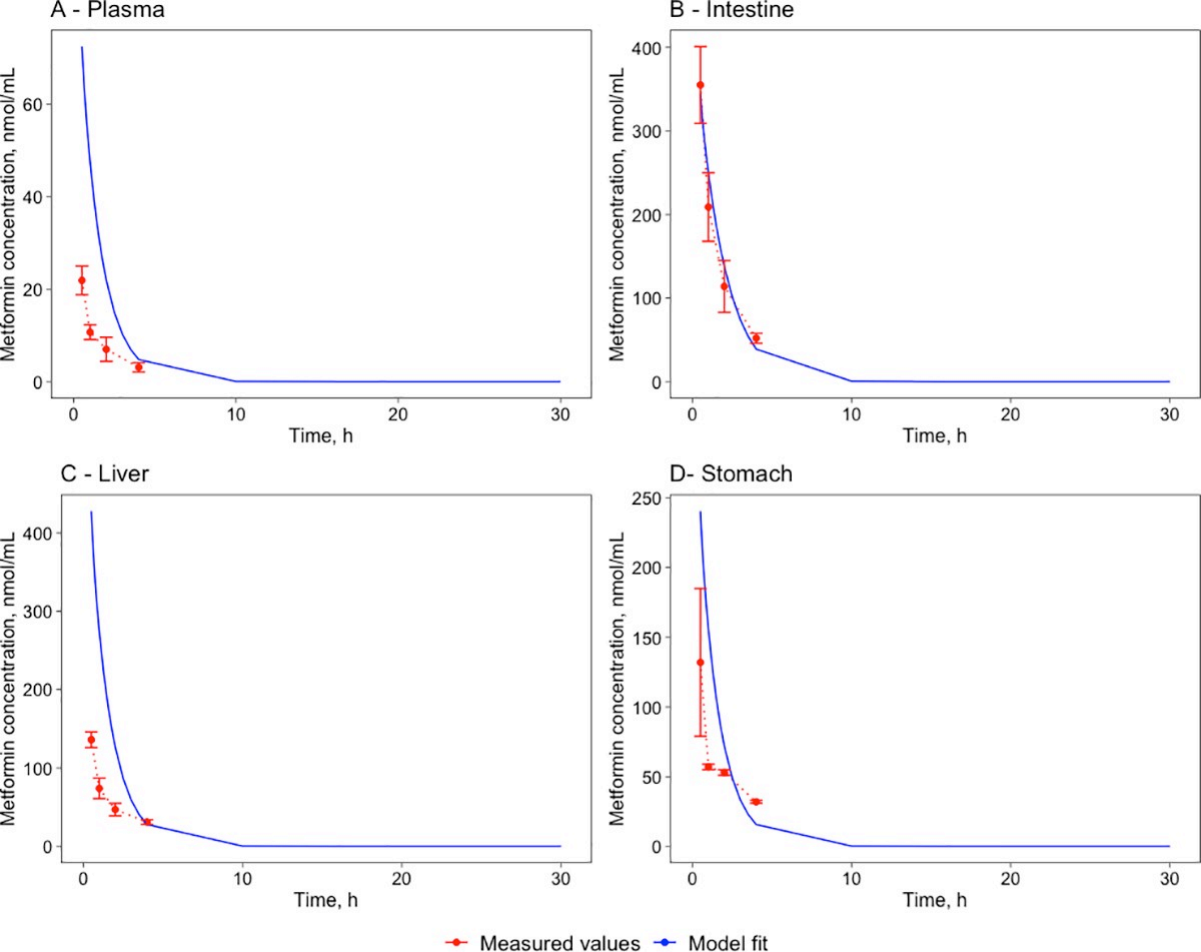
Metformin pharmacokinetics. Venous plasma (A), small intestine (B), liver (C), and stomach (D) following a single intravenous 50 mg/kg dose in mice. The red marks represent the experimental data’s concentration-time profiles with error bars representing standard deviation [1] and the blue lines represent the model simulations.

A mathematical model for mice has been developed (see Methods and **S1 Text, S1 Data,** and **S1 Table** for details). The mice models simulating single per-oral dose (BioModels ID: MODEL2103020001) and single intravenous dose (BioModels ID: MODEL2103020002) were deposited in *BioModels* data base [34] in SBML L2V4 format and as COPASI files. The model parameter estimation of the plasma-tissue partition coefficients (K_t:p_) was performed simultaneously using both the intravenous (IV) dataset, including plasma, small intestine, and stomach measurements, as well as peroral (PO) dataset, including plasma, portal vein, small intestine, liver, kidney, heart, muscle, fat, and brain from Wilcock experiments [1] of a 50mg/kg metformin dose. All parameters of the mice model are presented in the supplementary file **S1 Table**. The estimated parameters were K_t:p_ and V_max_ values, while physiological parameters were taken from literature data (see section 4.2.2). A single set of parameter values was obtained for both the IV and the PO experimental datasets. The experimentally determined concentration-time profiles were compared with the model simulations in Fig 1 for the PO and Fig 2 for the IV administration. The mice model was not validated due to the lack of appropriate experimental data.

#### 2.1.2. Pharmacokinetic parameters

Key pharmacokinetic parameters–area under the curve (AUC_24_), half-life (T_1/2_), maximal concentration (C_max_), and time of maximal concentration (T_max_) were compared (Table 1) to evaluate the predictive capability of the model. The parameters were calculated for both the experimental and model simulations following a 50mg/kg dose. Due to the IV curves’ decreasing nature, only AUC_24_ and T_1/2_ values were calculated for the IV experimental data (Table 2).

**Table 1:** Metformin pharmacokinetic parameter comparison of experimental data [1] and model simulations in plasma, portal vein, intestine, liver, kidney heart, muscle, adipose tissues, and brain following a single 50mg/kg PO dose in mice.

Tissue	Type	Cmax. nmol/mL	Cmax measured -fitted. %	Tmax. h	Tmax. measured-fitted. %	AUC24. nmol*h/mL	AUC measured- fitted. %	T1/2. h	T1/2 measured- fitted. %
Plasma	Measured	29.0	-6	0.5	150	160.8	-35	3.6	-31
	Fitted	27.2		1.3		105.3		2.5	
Portal vein	Measured	52.0	-22	0.5	50	217.6	-34	2.1	16
	Fitted	40.7		1.8		142.9		2.4	
Intestine	Measured	1971.0	-17	1.0	0	7291.6	-22	1.7	35
	Fitted	1636.5		1.0		5652.8		2.3	
Liver	Measured	253.0	-14	0.5	75	1112.9	-31	2.1	15
	Fitted	216.6		0.9		768.7		2.4	
Kidney	Measured	428.0	-2	0.5	150	1541.3	4	0.9	180
	Fitted	420.7		1.3		1603.1		2.5	
Heart	Measured	70.0	-3	0.5	150	236.7	11	2.4	4
	Fitted	68.1		1.3		263.2		2.5	
Muscle	Measured	102.0	5	2.0	-13	501.0	-14	3.3	-27
	Fitted	107.3		1.8		431.2		2.4	
Adipose	Measured	19.0	0	2.0	-13	93.8	-18	3.1	-22
	Fitted	19.1		1.8		76.8		2.4	
Brain	Measured	18.0	21	4.0	-69	107.7	-22	2.2	21
	Fitted	21.8		1.3		84.2		2.7	

**Table 2:** Metformin pharmacokinetic parameter comparison of experimental data [1] and model simulations in plasma, intestine, and stomach following a single 50mg/kg intravenous dose in mice.

Tissue	Type	T_1/2_, h	T_1/2_ measured- fitted, %	AUC_24_, nmol*h/mL	AUC measured- fitted, %
Intestine	Measured	0.8	46	452.5	21
	Fitted	1.2		549.5	
Plasma	Measured	0.5	72	26.1	250
	Fitted	0.9		91.4	
Stomach	Measured	1.0	-13	182.9	190
	Fitted	0.9		529.5	
Liver	Measured	0.7	17	191.2	173
	Fitted	0.8		523.5	

Generally, the pharmacokinetic parameters have a good agreement between experimental and simulated curves. There are discrepancies in the T_max_ values between the experimental data and the model simulations for the PO dataset. That is partly due to the sampling frequency in the experimental dataset, where the maximum may be situated between the sample collection time points (e.g., 0.5; 1; 2; 3; 8 and 24h). The most significant T_max_ discrepancies are in the plasma, heart, liver, kidney, and brain tissues. The differences in the pharmacokinetic parameters for the portal vein compartment are in the C_max_ and AUC_24_ value,s where C_max_ is lower by 22% in the model simulations, causing the difference in the AUC_24_ by 34%. The model can simulate the dynamic tendencies of the portal vein concentration-time profile. Differences are also noticeable in the intestinal compartment, where the AUC_24_ and T_1/2_ values are lower for the model simulations by 22% and 35% correspondingly. These differences can be expected as the intestinal kinetic parameters are scaled from Proctor [35] where experiments are carried out in cell cultures. Still, the kinetic parameters could differ under physiological circumstances. The kidney’s differences are in the T_1/2_ values, where 0.9h measured vs. 2.5h simulated leads to a 180% difference (just 1.6h in absolute numbers). The underlying cause of this difference is that the parameters concerning active excretion of metformin–parameters were estimated by simultaneously running simulations for the PO and IV experiments. Since the IV dataset elimination is much quicker than in the PO dataset, the resulting active excretion parameters are a compromise between the two. Differences in the C_max_ values for metformin concentration in the brain tissues could indicate a more complex transport mechanism across the blood-brain barrier that is not depicted in the model. On the other hand, the brain tissue concentration time-curve shows a surprising concentration fall at the 2^nd^ hour and an increase at the 5^th^ hour–this is uncommon for metformin concentration-time profiles as they usually have only one peak for a single-dose regimen. A similarly unusual fall in the concentration-time shapes can be noticed in the venous plasma and portal vein experimental concentration-time curves.

The differences between the model simulations are more extensive for the IV dataset than in the PO dataset due to rapid excretion. In the model simulations, metformin is excreted slower than in experimental results. Therefore the AUC_24_ and the T_1/2_ values are more significant than in the literature dataset, but the curve dynamics are very similar (see Fig 2). The biggest difference can be observed in plasma: T_1/2_ is measured at 0.50h while model simulations show 0.9h giving a 24-minute delay. The even bigger difference is in the AUC values: the measured value is 26.1 nmol*h/mL, and the simulated value is 91.4 nmol*h/mL. The differences in both cases are caused by a delayed concentration decrease in plasma as the model simulations are required to create a single compromise parameter set by considering the stomach and experimental intestine concentrations and the PO dataset.

The resulting plasma tissue partition coefficients for the various tissues are combined in Table 3. The estimated K_t:p_ parameters (see section 4.4.2) are similar to the calculated, with the largest difference of 82.5% being in the adipose tissues and 60% difference in the brain, and 53% in intestinal tissues.

**Table 3:** A comparison of metformin tissue-plasma partition coefficients in various mice tissues calculated using the method from Rogers et al. [14] and estimated in the model using parameter estimation, n.a.–data not available.

Tissue	Calculated K_t:p_	Estimated K_t:p_	Calculated–estimated, %
Intestine	3.0	4.6	53.3
Stomach	n.a.	3.2	n.a.
Liver	7.0	5.5	-21.4
Lungs	2.7	3.0	11.1
Brain	2.0	0.8	-60.0
Muscle	4.9	4.1	-16.3
Adipose	0.4	0.7	82.5
Heart	3.1	2.5	- 19.3
Remainder	n.a.	0.8	n.a.
Kidney	4.3	4.5	4.7

### 2.2. Metformin PBPK model in human

#### 2.2.1. Parameter estimation

A mathematical model for humans has been developed (see Methods and **S1 Text, S1 Data,** and **S2 Table** for details). The human models simulating single per-oral dose (BioModels ID: MODEL2103020003) and multiple per-oral dose with eight PO administrations with 12h interval (BioModels ID: MODEL2103020004) were deposited in *BioModels* data base [34] in SBML L2V4 format and as COPASI files. The mice model was scaled-up to represent the human body (mainly tissue volumes and blood flow values) described in section 4.5. The human model’s scaling coefficients were determined using parameter estimation and resulted in 0.7 for the absorption and 320 for the elimination processes. The complete set of the human model parameters is combined in the supplementary **S2 Table**. The model simulations were fit to two experimental datasets simultaneously to produce a single parameter set. The experimental data sets used included concentrations in plasma, red blood cells, and urine from Zaharenko [20] for a 500mg PO dose of metformin hydrochloride and the experimental data of concentrations in plasma for a 1000mg dose from Chung [36] (see section 4.3.2).

**Fig 3:**
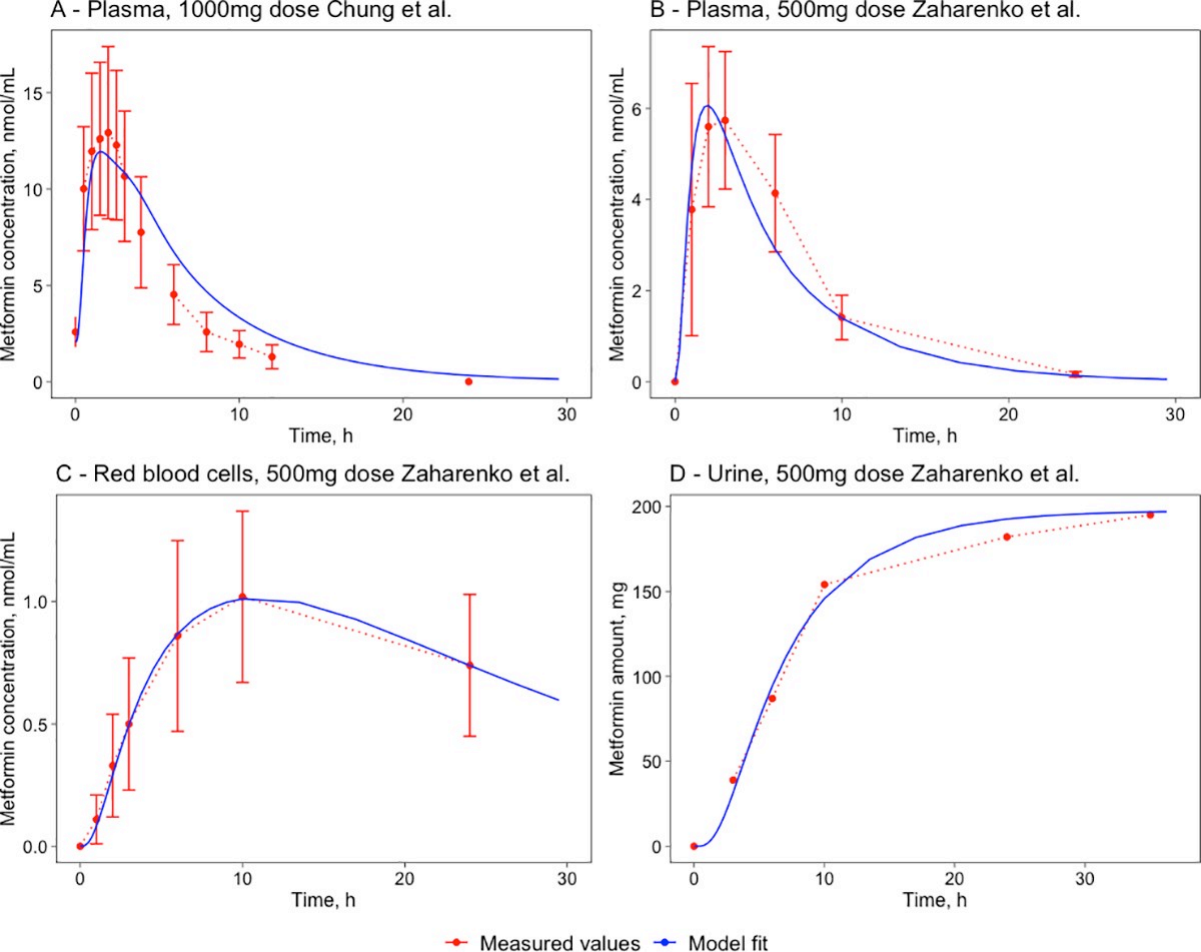
Metformin time-course comparison of experimental data and model simulations for tissues. Plasma (A) following a single 1000mg PO dose in humans [36] and simulations for plasma (B), red blood cells (C), and urine (D) following a single 500mg PO dose in humans [20]. The red marks represent the experimental data’s concentration-time profiles, where the red error bars represent the standard deviation, and the blue lines represent the model simulations.

The model simulations have a good fit to the experimental data and represent the experimentally observed concentration-time profiles in humans (Fig 3). The model simulations are also able to describe the proportions of metformin excretion in urine and feces. The comparison of the pharmacokinetic parameters of model simulations and experimental data are combined in Table 4. For most parameters, the differences are within a 30% range except in the 1000mg dose when there is a 44.1% difference between the metformin plasma T_1/2_ parameters—this is due to the model simulations compromising between the two datasets that have very different terminal half-life values.

**Table 4:** Metformin pharmacokinetic parameter comparison of experimental data and model simulations in plasma and red blood cells following a single 500mg PO dose in humans [20] and simulations for plasma following a single 1000mg PO dose in humans [36].

Study	Tissue	Type	C_max_, nmol/mL	C_max_ measured- fitted, %	T_max_, h	T_max_ measured—fitted, %	AUC_24_, nmol*h/mL	AUC measured- fitted, %	T_1/2_, h	T_1/2_ measured-fitted, %
Zaharenko, 500mg	Plasma	Measured	5.7	7.0	3	-33.3	45.1	-6.4	4.9	-24.3
		Fitted	6.1		2		42.2		3.7	
	Red blood cells	Measured	1.0	0.0	10	0.0	18.7	3.2	21.0	12.3
		Fitted	1.0		10		19.3		23.6	
Chung 1000mg	Plasma	Measured	12.9	-2.3	2	-10.0	74.1	17.0	2.7	44.1
		Fitted	12.6		1.8		86.7		3.9	

#### 2.2.2. Validation results

**Fig 4:**
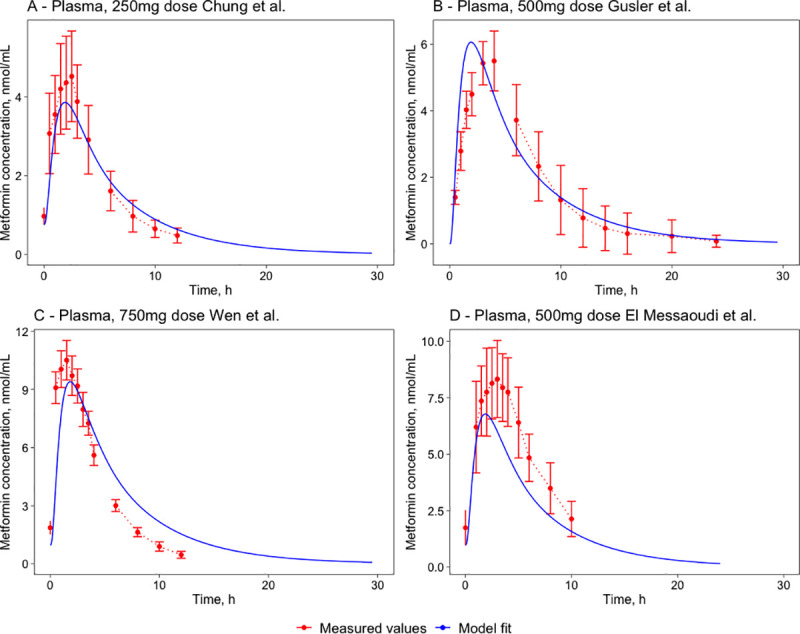
Metformin pharmacokinetics in plasma—model validation results for 250mg (A) [36], 500mg (B) [37], 750mg (C) [38] and 500mg (D) [39] PO doses in humans. The red marks represent the experimental data’s concentration-time profiles from four different datasets, the error bars represent standard deviation, and the blue lines represent the model simulations.

Without changing any parameters except for the dose, the human model was validated against four separate datasets (see section 4.3.2) with different PO doses– 250mg dose dataset from Chung [36] with one 375mg pre-dose 12 hours before the main dose, 500mg dose dataset from Gusler [37] without a pre-dose, 750mg dose dataset from Wen [38] with one 500mg pre-dose 12 hours before the main dose and a 500mg multiple dose dataset from El Messaoudi [39] with six pre-doses of 500 mg with a time interval of 12h. Model simulations were compared to experimental datasets, and model simulations can predict metformin pharmacokinetics at varying doses and multiple dosing regimens (Fig 4).

Critical pharmacokinetic parameters were also compared between the datasets, and the model predictions and results are combined in Table 5. The difference between model predictions and experimental data is <40% for most parameters, except for the discrepancies for the half-life values where there is a 56.5% increase in comparison with the Chung 250mg dataset and a 44.4% increase in comparison with the Wen 750mg dataset.

**Table 5:** Validation results of plasma metformin concentration pharmacokinetic parameters–a comparison of model predictions and experimental results of four different datasets in humans.

Study	Type	C_max_, nmol/mL	C_max_ measured—fitted, %	T_max_, h	T_max_ measured—fitted, %	AUC_24_, nmol*h/mL	AUC measured—fitted, %	T_1/2_, h	T_1/2_ measured—fitted, %
Chung 250mg	Measured	4.5	-13.3	2.5	-24.0	26.8	0.4	2.3	56.5
	Fitted	3.9		1.9		26.9		3.6	
Gusler 500mg	Measured	5.5	10.9	3.5	-42.9	39.6	6.6	3.9	-4.9
	Fitted	6.1		2.0		42.2		3.7	
Wen 750m	Measured	10.5	-10.5	1.5	26.7	51.9	25.0	2.7	44.4
	Fitted	9.4		1.9		64.9		3.9	
El Messaoudi 500mg	Measured	8.3	-18.0	3.0	-36.7	63.6	-24.4	4	-2.5
	Fitted	6.8		1.9		48.1		3.9	

### 2.3. Simulations of concentration-time profiles in humans

#### 2.3.1 Dose-dependent distribution in tissues

**Fig 5:**
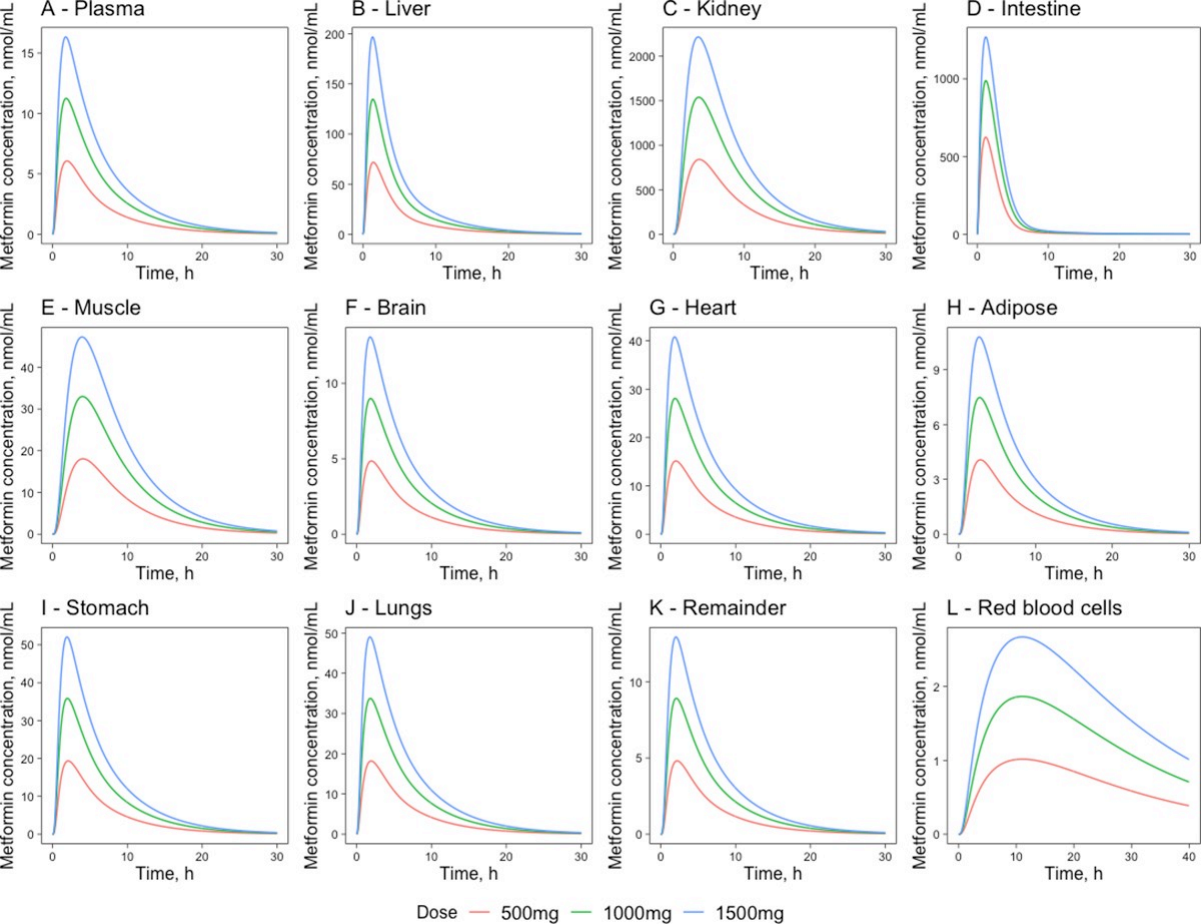
Metformin concentration time-courses in major compartments of metformin action. Plasma (A), liver (B), kidney (C), intestine (D), muscle (E), brain (F), heart (G), adipose (H), stomach (I), lungs (J), the remainder (K), red blood cells (L)—following a single PO dose of 500mg, 1000mg and 1500mg metformin hydrochloride in humans. The red lines represent the concentration-time profiles of the model simulations of the 500mg dose, the green lines represent model simulations for the 1000mg dose and the blue lines represent model simulations for the 1500mg dose.

The simulations were made for a single PO dose of 500mg, 1000mg, and 1500mg metformin hydrochloride, which corresponds to 389.2mg, 778.4m,g, and 1167.6mg metformin (see section 4.3). Concentration-time profiles for the tissues are combined in Fig 5. Pharmacokinetic parameters were also calculated for the simulated data and are incorporated in Table 6. The simulations show that the largest concentrations are reached in the intestinal and kidney tissues. The kidney tissues get a maximum concentration of 840 nmol/mL for a 500mg dose. The kidney concentration is greater than the concentration in the small intestine known to accumulate metformin. This could be because the kidney is the only organ responsible for metformin elimination, and in the human organism are a smaller proportion of the body than in the mice causing a higher metformin concentration. Most tissues have a half-life similar to plasma, except for the liver and intestine tissues that have a shorter T_1/2_ and muscle, kidney, and RBC that have a longer T_1/2_. The model simulations also show a dose-dependent absorption of metformin. After a 500mg dose, 50.7% of it (197.1mg) is excreted in the urine, after a 1000mg dose, 46.3% of the dose (362mg) is excreted, but after a 1500mg dose 44.1% (517mg) is excreted in the urine. That corresponds to a dose-dependent absorption with a higher fraction of the drug being absorbed at low doses [9, 19, 40]. The concentration-time profiles in organs that were not covered by experimental data were simulated using the K_t:p_ parameters determined from the mice model.

**Table 6:** Metformin pharmacokinetic parameters for different tissues following a single 500mg, 1000mg, and 1500mg PO dose in humans.

Tissue	Dose, mg	AUC_24_, nmol*h/mL	C_max_, nmol/mL	T_max_, h	Amount at C_max_, mg	T_1/2_, h
Plasma	500	42.2	6.1	2.0	2.3	3.7
	1000	76.1	11.2	1.9	4.3	3.7
	1500	109.0	16.3	1.8	6.3	3.7
Red blood cells	500	19.3	1.0	11.1	0.3	22.4
	1000	35.5	1.9	11.0	0.5	22.4
	1500	50.9	2.7	11.0	0.8	22.5
Adipose	500	30.2	4.1	2.8	7.9	4.2
	1000	55.4	7.5	2.7	14.5	4.2
	1500	79.4	10.8	2.7	21.0	4.2
Brain	500	33.2	4.9	2.0	0.9	3.8
	1000	60.9	9.0	1.9	1.6	3.8
	1500	87.2	13.1	1.8	2.4	3.9
Remainder	500	33.1	4.8	2.2	10.0	3.9
	1000	60.8	8.9	2.1	18.5	3.9
	1500	87.1	12.9	2.0	26.9	3.9
Muscle	500	168.4	18.1	4.1	65.2	5.5
	1000	309.3	33.0	4.0	119.2	5.6
	1500	442.9	47.2	3.9	170.7	5.7
Intestine	500	1 879.0	623.6	1.2	57.4	1.8
	1000	3 156.0	987.6	1.2	91.0	1.9
	1500	4 174.6	1 268.9	1.2	116.9	2.0
Lungs	500	124.3	18.2	1.9	1.2	3.9
	1000	228.4	33.7	1.8	2.3	3.9
	1500	327.0	49.0	1.8	3.4	3.8
Stomach	500	132.6	19.3	2.1	0.4	3.8
	1000	243.5	35.9	2.0	0.7	3.9
	1500	348.6	52.1	1.9	1.0	3.9
Heart	500	103.6	15.2	2.0	0.7	3.9
	1000	190.2	28.1	1.9	1.3	3.9
	1500	272.4	40.8	1.8	1.8	3.9
Liver	500	346.6	71.8	1.4	16.7	2.5
	1000	636.4	134.7	1.4	31.3	2.4
	1500	910.9	196.9	1.3	45.7	2.4
Kidney	500	7 235.1	840.0	3.7	33.2	5.0
	1000	13 302.6	1 539.2	3.6	60.9	5.0
	1500	19 087.0	2 216.9	3.5	87.7	5.1

**Fig 6:**
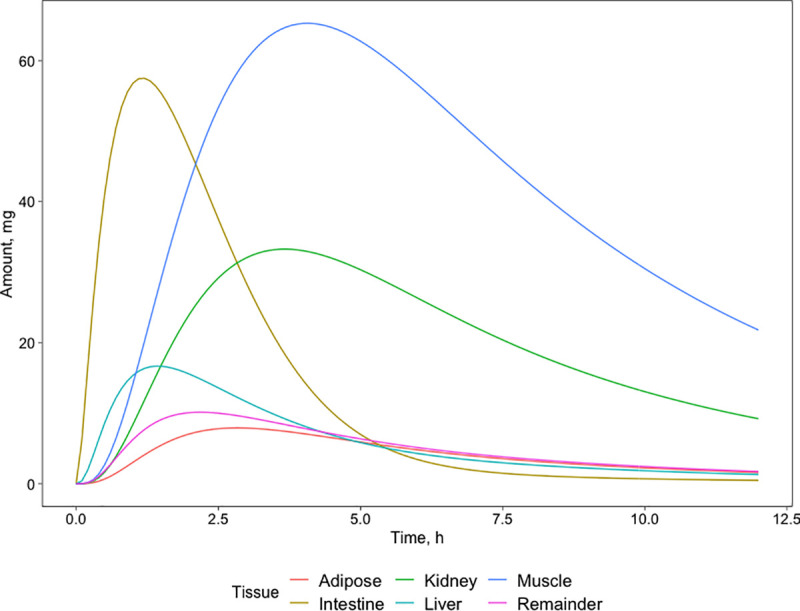
Distribution of metformin amount over tissues that contain more than 2% of the absorbed amount of metformin at PO dose 500 mg. Red color curves represent adipose tissues, green–kidney, dark blue- muscle, yellow–intestine, light blue–liver, pink–remainder.

In parallel to the tissues’ concentrations, an interesting aspect is distribution through the body in time derived by multiplying the metformin tissue concentrations with the volume of the tissues (Fig 6). In the case of a 500mg dose of metformin, hydrochloride 197 mg of metformin are absorbed. It turns out that no body fluid at its maximum concentration contains more than 2% of absorbed metformin. At maximum the metformin amounts reach different fractions of absorbed metformin: 33% (66mg) in muscle 29% (58 mg) in intestine, 17% (34mg) in kidney, 8.5% (17mg) in liver, 5% (10mg) in remainder and 4% (8mg) in adipose. The metformin concentration peaks of other tissues are below 2%.

#### 2.3.2. Multi-dosing regimen

**Fig 7:**
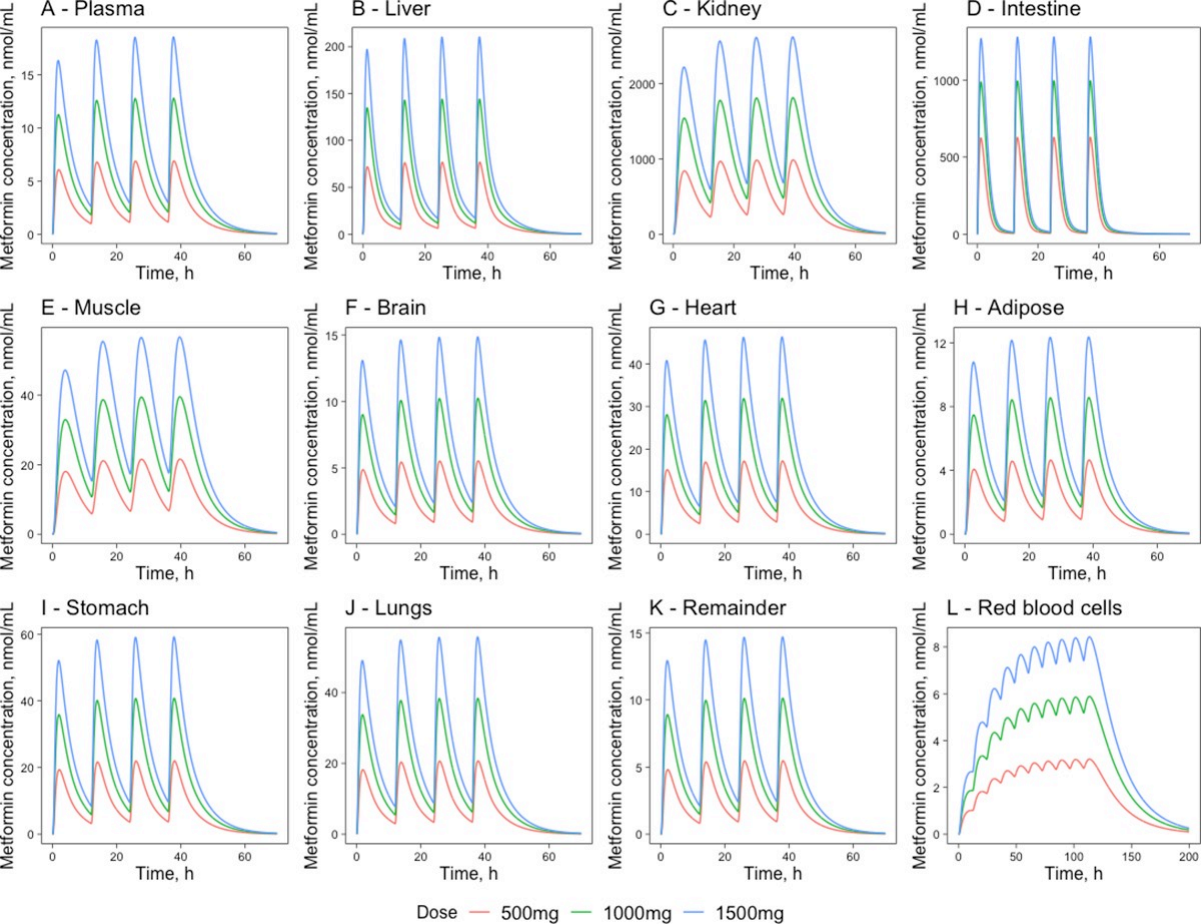
Metformin concentration time-courses in major compartments of metformin action. Plasma (A), liver (B), kidney (C), intestine (D), muscle (E), brain (F), heart (G), adipose (H), stomach (I), lungs (J), the remainder (K), red blood cells (L)—following four PO doses of 500mg, 1000mg and 1500mg in humans at 0, 12, 24 and 36h in humans. The red lines represent the concentration-time profiles of the model simulations of the 500mg dose, the green lines represent model simulations for the 1000mg dose and the blue lines represent model simulations for the 1500mg dose.

In the simulated multi-dosing regimen, frequently used metformin hydrochloride doses of 500mg, 1000mg, and 1500mg [9] were administered twice-daily every 12 hours (Fig 7). The key pharmacokinetic parameters are summarized in Table 7. The model simulations show that in the metformin concentrations, steady-state is reached after the third dose (around 24 hours) for all tissues except the red blood cells. The simulations also show that metformin is accumulated in the red blood cells. The maximal concentrations are two times higher under the multiple dosing regimen. A steady-state in the concentration is reached after 113.5 hours or approximately five days of twice-daily dosing. Under the multi-dosing regimen, the maximal concentrations reached in plasma are higher–for a single PO dose of 500 mg and 1000 mg metformin hydrochloride concentrations in plasma reach a maximum of 6.1 nmol/mL (0.79 mg/L) and 11.2 nmol/mL (1.45 mg/L) while in a multi dosing regimen the C_max_ value is 6.9 nmol/mL (0.90 mg/L) and 12.8nmol/mL (1.66 mg/L) respectively. The average steady-state concentration in plasma during a multi-dosing regimen is 3.5 nmol/mL (0.45 mg/L) for a 500 mg dose and 6.5 nmol/mL (0.84 mg/L) for a 1000mg dose. These results are comparable to those observed experimentally where the C_max_ after twice-daily dosing of 1000 mg is 1.32 ± 0.23 mg/L in health subjects [41], while the average plasma concentration at steady state is 0.86 ± 0.19 mg/L [42]. Since in a clinical setting metformin hydrochloride is not prescribed as a single PO dose but is regularly used multiple times per day, the developed model can be used to simulate such a dosing regimen.

**Table 7:** Metformin pharmacokinetic parameters for different tissues following a twice-daily dosing regimen of 500mg, 1000mg, and 1500mg PO dose in humans.

Tissue	Dose, mg	AUC_24_, nmol*h/mL	C_max_, nmol/mL	C_mean_, nmol/mL	Amount at C_max_, mg	T_max_, h	T_1/2_, h
Plasma	500	84.2	6.9	3.5	2.7	25.9	3.9
	1000	154.6	12.8	6.5	4.9	25.8	3.9
	1500	221.4	18.5	9.2	7.2	25.7	3.9
Red blood cells	500	90.3	3.2	3.1	0.9	113.5	21.7
	1000	166.1	5.9	5.6	1.7	113.5	22.1
	1500	238.7	8.4	8.0	2.4	113.5	22.1
Adipose	500	61.4	4.6	2.6	9.0	26.7	4.3
	1000	112.8	8.6	4.7	16.6	26.6	4.3
	1500	161.6	12.3	6.7	24.0	26.6	4.3
Brain	500	67.4	6.9	2.8	1.2	25.9	4.0
	1000	123.7	12.8	5.2	2.3	25.8	4.0
	1500	177.2	18.7	7.4	3.4	25.7	4.0
Remainder	500	67.3	5.5	2.8	11.3	26.1	4.0
	1000	123.8	10.1	5.2	21.0	26.0	4.0
	1500	177.1	14.7	7.4	30.6	26.0	3.9
Muscle	500	334.7	21.6	14.4	77.9	27.8	5.5
	1000	633.1	39.5	26.4	142.6	27.7	5.6
	1500	906.7	56.6	37.9	204.6	27.6	6.1
Intestine	500	3 764.0	627.8	156.9	57.8	25.2	1.8
	1000	6 320.1	995.1	263.4	91.7	25.2	2.0
	1500	8 358.7	1 279.3	348.3	117.8	25.2	2.0
Lungs	500	252.6	20.6	10.5	1.4	25.9	3.9
	1000	463.9	38.3	19.4	2.6	25.8	3.9
	1500	664.3	55.6	27.7	3.8	25.7	4.0
Stomach	500	269.4	21.9	11.2	0.4	26.0	4.0
	1000	494.8	40.7	20.6	0.8	25.9	4.0
	1500	708.6	59.0	29.6	1.1	25.9	3.9
Heart	500	210.5	17.2	8.8	0.8	25.9	4.0
	1000	386.6	31.9	16.1	1.4	25.8	4.0
	1500	553.6	46.3	23.1	2.1	25.8	3.9
Liver	500	700.4	76.7	29.2	17.8	25.4	2.6
	1000	1 285.8	143.8	53.6	33.4	25.3	2.6
	1500	1 840.6	210.1	76.8	48.8	25.3	2.4
Kidney	500	14 777.7	983.1	616.9	38.9	27.5	5.0
	1000	27 185.9	1 806.9	1 134.8	71.5	27.4	5.0
	1500	39 059.5	2 610.3	1 630.4	103.2	27.3	5.1

#### 2.3.3. Simulations of tissue proportion impact on pharmacokinetics

The impact of an individual’s specific tissue distribution can be taken into account in metformin pharmacokinetic simulations. The simulations were performed to find the impact of specific tissue proportions with 1) an increased muscle volume to model a sportsman and 2) an increased adipose tissue volume to model obesity. In both cases, the blood flow through the muscles and the adipose tissues was increased proportionally to the tissue, thus creating a greater cardiac output.

**Fig 8:**
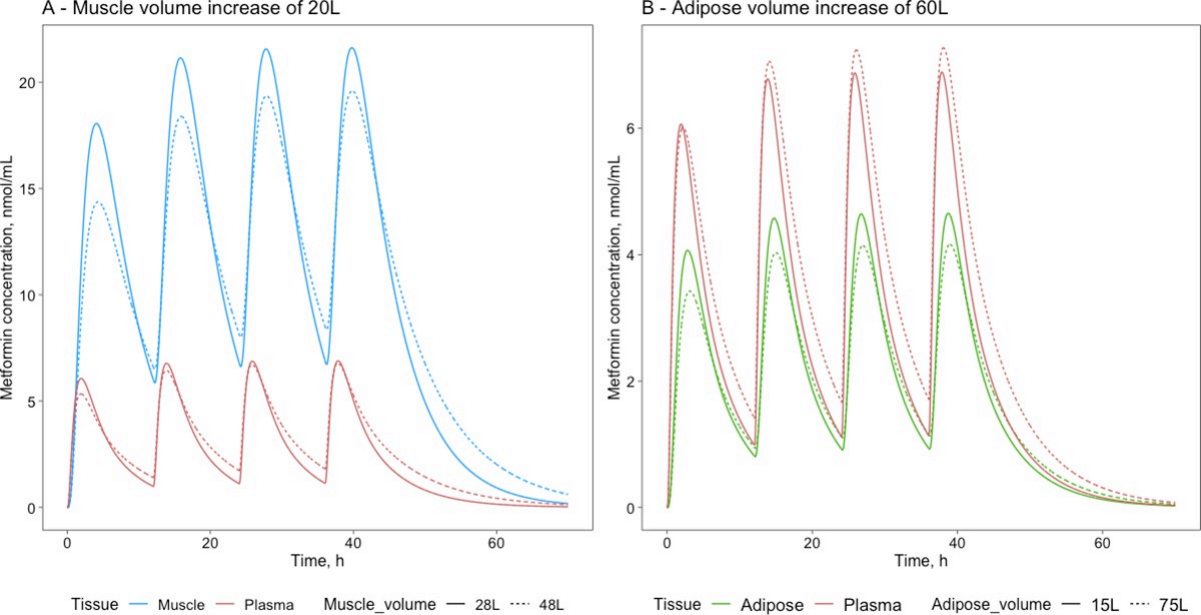
Metformin concentrations in A—muscle and plasma at normal muscle volume (28L) and increased muscle volume 48L and B—adipose and plasma at normal adipose volume (15L) and increased adipose volume 75L.

For the sportsman simulation, when muscle tissue volume was increased by 20L (+72% compared with the initial volume of 28L) (Fig 8A), metformin concentration oscillation amplitude reduces by 28%, while the average concentration decreases just by 2.5%. There is no decrease of the maximum concentration in plasma and an increase by 20% of the concentration at the minimum point. For the obesity simulation (Fig 8B), adipose tissue volume was increased by 60L (+ 400% compared with the initial volume of 15L), the average metformin concentration at a steady-state in adipose tissues decreased by 7%, and the oscillation amplitude was decreased by 25%. At the same time, plasma oscillation amplitude decreased by 2%, and average concentration increased by 10%. These different muscle and adipose trends can be explained by the differences in their K_t:p_ values (4.9 for muscle and 0.4 for adipose, see Table 3).

## 3. Discussion

Due to the large differences in the pharmacokinetic profiles and the therapeutic outcomes of metformin, it is desirable to gain insight into its concentration in various tissues. Physiologically based pharmacokinetic (PBPK) modeling is considered to be the most comprehensive pharmacokinetic modeling used to describe the pharmacokinetics of active pharmaceutical ingredients of interest. Several metformin PBPK models have been developed to describe the transporter-mediated drug-drug interaction and the pharmacokinetics in special populations [8, 28–33]. As opposed to other PBPK models in our approach, we assume the transport processes in humans to be similar to those in mice, and transferable Kt:p values integrate both the active and passive components of metformin transport, which are tissue-specific, and are transferable among different species. This allows the determination of metformin concentration-time profiles in major compartments of metformin action. The prediction of metformin exposure in various tissues gains additional importance as the metformin has demonstrated the possible beneficial potential of metformin administration for a wide range of pathological conditions [6, 7] where the pharmacodynamic targets are not fully identified, so concentration-time series in target tissues could allow making further assumptions and predictions. The model was also individualized to simulate obesity and an increase in muscle mass. Several similar scale-up studies have been done before, and it seems to be the best alternative to shed light on the pharmacokinetics of humans in the case of a lack of information about the concentration-time profiles in human tissues [24, 43–45].

### 3.1. PBPK model for mice

For all the tissues, model simulations of the PO dose closely mirrored the experimental concentration-time data, while higher discrepancies were observed for the fit between the IV dose experimental data and model simulations. It was considered as acceptable as the establishment of the PO route is the main topic in this study, while simulations of the IV route replicated the character of curves with acceptable accuracy. Thus, one can conclude that the model does not replicate metformin elimination mechanisms at very high plasma concentrations as in IV experiments. It is in line with described flip-flop PK of PO administrated metformin [11, 35, 46], as the rate of absorption is slower than that of elimination. Considering that clearance of metformin is comparable between species, it could be assumed that clearance after PO administration is about four times lower compared to that after IV administration [42, 47].

The estimated K_t:p_ coefficients describe the passive and active transport [27]. The estimated values were compared with the values that were calculated based on the prediction method from Rodgers et al. [14]. The comparison of the K_t:p_ coefficients demonstrated discrepancies between the estimated and the calculated values (Table 3). However, the demonstrated discrepancies can be explained by the active transport fraction, which was not taken into account during calculation [14, 27]. The active transport in skeletal muscle, liver, and especially in the small intestine is very influential, and the differences between the estimated and calculated K_t:p_ coefficients are expected [42]. The higher differences between the estimated and calculated K_t:p_ in the brain could be explained with the oversimplification of the brain model since it is expected that the brain penetration is more restrictive and selective compared to other tissues. Besides, it is described that OCT3 is expected to be involved in metformin transport over the blood-brain-barrier [48]. Additionally, it could be expected that the experimental data of mice do not appropriately describe C_max_ of metformin in brain tissue due to a local minimum of concentration at two h.

The small intestine related reactions have been adapted from Proctor [35] with the exception of the V_max_ value of the reaction “03.3. Enterocytes -> IntestineVascular (OCT1)” where the V_max_ value was reduced from 2376nmol/ml according to Proctor to 495nmol/ml to enable a better fit with the experimental dataset. That is in accordance with the comments from Proctor stating that this reaction is not actively used due to its high K_m_ value. In the model simulations, however, reached concentrations are much higher than in the experimental setup of Proctor [35].

### 3.2. PBPK model for humans

The extrapolated model demonstrates a good fit with the dataset from Chung with a single PO dose of 1000 mg [36] and the datasets from Zaharenko [20] with a single PO dose of 500 mg, which were used for the development of the human PBPK. The model predictions at different dose amounts and regimens were compared with four independent datasets of plasma metformin concentrations to validate the human model. The model predictions of metformin maximum plasma concentration (C_max_) are considered accurate for the single 250 mg and 500 mg PO doses of metformin hydrochloride and for the multiple 500 mg and 750mg doses of metformin hydrochloride [36–39]. Subject populations of these four studies were comparable, as there were no significant differences in the physiological characteristics like age, body mass index, or health status.

#### 3.2.1. Absorption

Metformin manifests a specific bio-availability profile due to its physio-chemical characteristics. Bio-availability for most drugs is determined by the absorption and metabolism in the gastrointestinal tract and hepatic metabolism, but as metformin is not metabolized in the human body, its bio-availability is determined primarily by intestinal absorption. Metformin pharmacokinetics are described by a slow absorption which is the main factor for its rate-limiting disposition through the body [19, 35]. In this model, an existing metformin absorption mechanism developed in cell cultures by Proctor et al. (2008) was used to describe absorption both in mice and humans.

The simulations show that T_max_ in the developed model is sensitive to the gastrointestinal transit time, which complies with the previously published data where it was demonstrated that metformin requires the entire length of the intestine to achieve its absorption ratio [49]. The developed model neglects the physiological division of the small intestine in different segments. As the particular absorption rate differences between these segments are unknown, it was assumed that the absorption takes place at the same ratio along the whole small intestine. However, possible differences in the permeability ratios across the small intestine segments cannot be excluded as the results from various authors are inconsistent. According to Song et al. (2006), the duodenum was found to be the main part of the gastrointestinal tract where absorption of metformin takes place. Respectively metformin is less actively absorbed in jejunum and ileum [50]. However, other authors emphasize the importance of other regions of the small intestine [49, 51]. Moreover, according to Vidon et al. (1988), only 20 percent of the PO dose can be absorbed in the duodenum. In further model development, the small intestine could be segmented and such differences could be introduced to understand metformin absorption better.

#### 3.2.2. Elimination

It has been described previously that metformin demonstrates negligible plasma protein binding. Due to that, it is expected that metformin should have a high unbound fraction in plasma [9], so part of metformin should be excreted in a fast manner by the glomerular filtration process. Some extended delay of metformin execration has been observed it is considered to be due to the slow distribution of metformin in the red blood cells [8], but because the concentration in the red blood cells is small and at T_max_ contains below 0.5% of the dose, it was not an impactful factor for the metformin excretion.

Since metformin is excreted unchanged through the kidney, metabolism was not introduced, and the liver was described as a well-stirred compartment. Metformin is exclusively eliminated through the renal pathway, and the renal clearance of metformin was found to be around 500 ml/min in humans [42]. A relatively high renal clearance indicates that the elimination mechanisms hugely rely on active transport. Active transporters could also have a high impact on the observed individual differences of metformin clearance in the population. The importance of the active transport role in metformin elimination has been supported by studies in the OCT1/OCT2-knockout mice, where the observed clearance decreased to approximately the unbound glomerular filtration rate [47]. It is expected that the active transport in renal clearance is primarily mediated by three types of transporters, OCT2, MATE1, and MATE2, which were introduced in the developed model to describe the secretion of metformin into the urine. The developed model describes the elimination of metformin acceptably and based on results (T_1/2_), it looks like the clearance is a little overestimated. It could be due to the underestimated accumulation in tubular cells (renal tissue compartment in the model), as there are some indications of possible active reabsorption from the tubular lumen [13]. Additionally, there could be differences in active transporter ratios among the different segments [13, 48] of the renal tubules, which were not incorporated in the developed model. It could be assumed that a genetic variation of the active renal transporters could have the highest clinical significance and importance for the therapy individualization, as it could be the main genetically variable factor having an impact on metformin exposure [13, 42].

#### 3.2.3. Precision medicine by model personalization

The model has two types of parameters—measurable parameters (weight, proportions of tissues, blood flow, clearance peculiarities) and those assessed using parameter estimation. Measurable parameters can be set in the model to correspond to a particular person. The unknown dynamic parameters can be estimated using the parameter estimation procedure if metformin concentration time-course data for plasma and urine after dose administration are recorded for a particular person. The deviations in transport protein concentrations can be mapped with genetic characteristics. Thus, generally, a personalized metformin PBPK model enables a precision therapy design to reach the therapeutic concentrations in tissues of interest, and precision medicine is the most promising application area of the proposed model. Despite the fact that some parameters are taken from mice, the developed PBPK model is an important improvement that considers the mechanistic interpretation of metformin pharmacokinetics compared to the current approach where only plasma and/or blood concentrations are taken into account for therapy development.

There are multiple limitations to the applicability of the model. Different individual peculiarities of metformin pharmacokinetics can compensate each other and are unidentifiable just from the measurements of metformin concentration time-profiles in blood and urine. As the data regarding the transporter activity in different tissues is missing, it is assumed in the model that the enzyme concentrations are constant and are not regulated depending on the concentration of metformin or individual genetics–the variance of these parameters could impact the individual response to metformin. Another issue is that the diet is not considered in the model and could be impactful on how metformin is absorbed and tolerated.

In summary, we have been able to develop a simplified PBPK model for metformin in humans, compensating for the lack of metformin concentration time-course data in tissues by applying some parameters from the mice model where tissue data was available for the human model parametrization. As a result, the dynamics of metformin distribution in humans can be predicted based on the information of the physiological properties of humans, the physio-chemical properties of metformin, and its excretion. The developed mathematical model can be applied for a particular patient by entering the proportions of tissues and parameters of blood flow and using data from a single dose experiment to record metformin concentration dynamics in blood and urine. The obtained concentration time-series after model parameter estimation for using the individual experimental data leads to a personally parametrized model that can be used as a decision support tool to develop individually tailored precision therapy to reach the necessary metformin concentrations in the target tissues. While it is possible to simulate the individual response to metformin, significant limitations to the model application exist. We acknowledge that this is a PBPK model that can be further improved with a greater understanding of metformin absorption, distribution, metabolism, and excretion from subsequent *in vitro* and *in vivo* studies.

## 4. Methods

### 4.1. Modeling software

Models were developed in *COPASI* (COmplex PAthway SImulator) simulation software [52, 53] version 4.27 (Build 217). The parameter estimation was conducted using *COPASI* built-in functions or estimated by fitting unknown model parameter values to experimental data using global stochastic optimization methods. The model-specific parameter estimation performance of global stochastic optimization methods implemented in *COPASI* were tested using *ConvAn* software [54]. Multiple parallel optimization runs were applied using *COPASI* wrapper *SpaceScanner* [55] to reduce misinterpretation risks of optimization results [56]. The various model parameters were either obtained from the literature, inferred from experimental data. Validation of these parameters is described in Results section.

The pharmacokinetic parameters (area under the curve (AUC_24_), half-life (T_1/2_), maximal concentration (C_max_), and time of maximal concentration (T_max_)) of the concentration-time curves were calculated using the *R package* (https://CRAN.R-project.org/package=PKNCA) version 0.9.4.

### 4.2. Physiologically Based Pharmacokinetic (PBPK) Models for mice and human

#### 4.2.1. The structure of models

**Fig 9:**
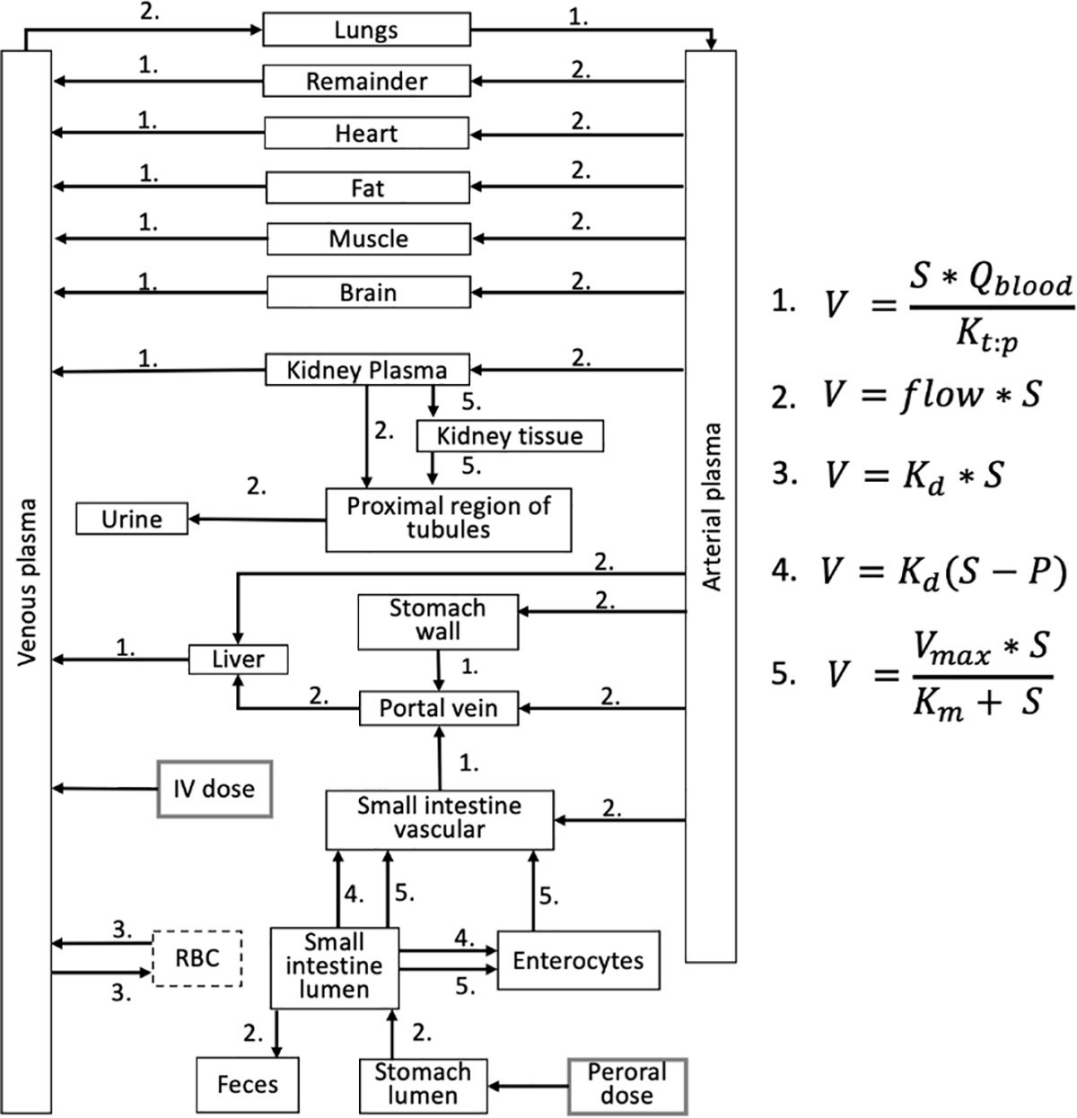
Schematic representation of a physiologically based pharmacokinetic model for metformin in mice and humans. **V–**the reaction rate**, S–**the concentration of metformin at substrate side**, P**- the concentration of metformin at product side, **Q**_**blood**_**−**the flow to a particular compartment**, K**_**t:p**_**—**tissue:plasma partition (Kt:p) coefficients, **K**_**d**_**—**the non-saturable component of transport**, V**_**max**_**—**the maximal velocity**, K**_**m**_**—**the Michaelis-Menten constant. Red blood cells (**RBC**) compartment (dashed line) is used only in the human model.

Generic PBPK models for mice and humans are built in the form of ordinary differential equations (Fig 9). The model structure is based on the PBPK compartment model reported by Aarons [23] where the remainder, heart, fat, muscle, brain, lungs, stomach, liver, portal vein, venous and arterial plasma, and red blood cells are defined as independent one compartment organs, while kidney and small intestine were developed as multicompartment organs to describe physiological processes by more detailed approach [23]. The concentrations in other organs such as the bone, skin, colon, etc., were not considered as separate compartments, and they are united in the model as a remainder compartment. An additional red blood cells (RBC) compartment was implemented in the human model structure (Fig 9) due to the fact that the concentration time-course data in red blood cells was available in humans [20] while it was missing for mice. There are no other structural differences between mice and human models. Tissue volumes and blood flow rates for the remainder compartment were calculated as the remaining fractions. Each compartment is associated with a blood flow rate, volume, and tissue partition coefficient. These compartments are interconnected by the arterial and venous plasma compartments. The developed model attempts to mimic and mathematically describe the dominating physical and biophysical processes that determine the pharmacokinetics of the metformin in the body.

**Fig 10:**
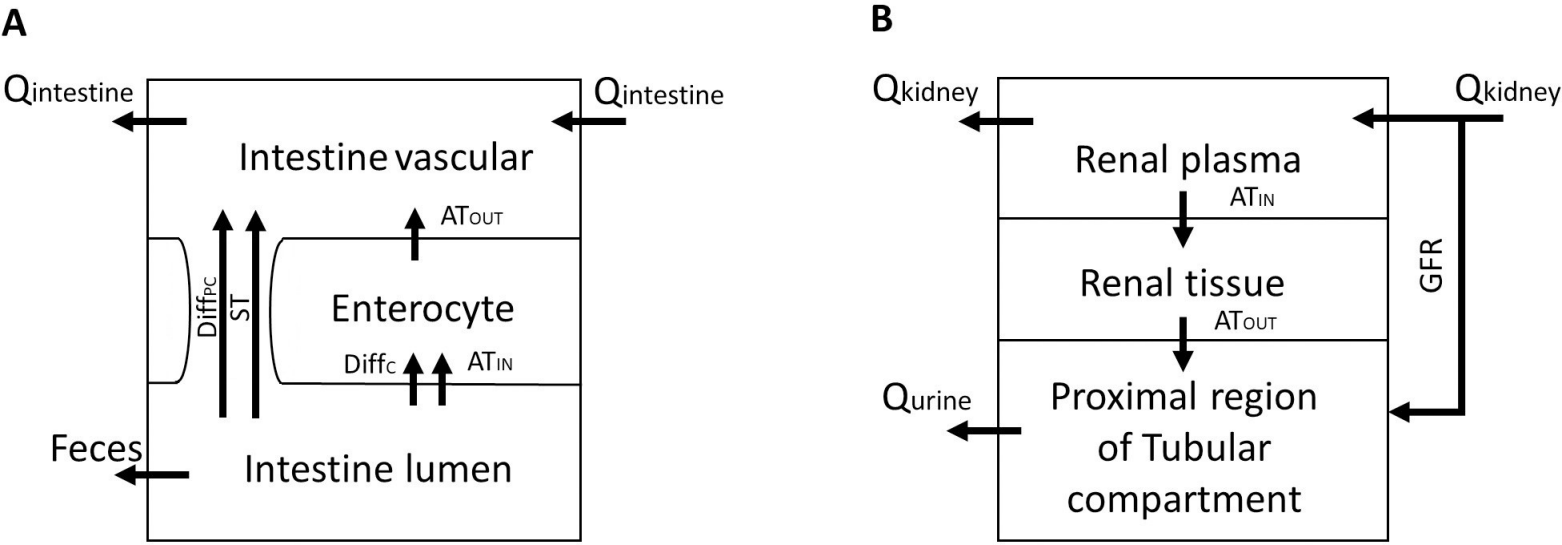
Intestine and kidney model structures for permeability rate-limited kinetics. **A:** Intestinal structure, where Q_intestine_−blood flow to the small intestine, ST—saturable transport through paracellular space, AT–active transport, Diff_c_−diffusion into cells, Diff_PC_−paracellular diffusion, AT_IN_−active transport into cells by OCT3 and PAMAT transporters, AT_OUT_—active transport out of cells by OCT1 transporters; **B:** Renal structure, where Q_kidney_−renal blood flow, Q_urine_−urine flow, GFR—glomerular filtration rate, AT_IN_−active transport into cells by OCT2 transporters, AT_OUT_—active transport out of cells by MATE1, MATE2-K and OCT1 transporters.

The model is developed assuming that all compartments except for the kidney (Fig 10A) and the small intestine (Fig 10B) are well stirred. It is assumed that the drug distribution into these compartments is driven by permeability-limited kinetics. The assumption of permeability-limited kinetics is based on the hydrophilic characteristics of metformin. Due to that, the active transport component is applied in the compartments of the kidney and small intestine of the developed model. Since the binding of metformin to the plasma proteins is negligible, the free concentration of metformin is expected to be equal to the total concentration of metformin [19, 57].

The processes describing metformin absorption via the small intestine and elimination via the kidney are described in detail as multiple compartment organs. The small intestine (Fig 10B) is divided into three compartments representing the blood and the interstitial space by the intestine vascular compartment, the enterocytes of the intestine wall are represented by the enterocyte compartment, and the lumen volume of the small intestine has been incorporated as the intestine lumen. The kidney (Fig 10A) is represented by three compartments, that respectively describe the plasma delivered to the kidney after glomerular filtration as renal plasma, the approximate volume of proximal tubules lumen as a compartment of the proximal region of the tubular compartment, and the rest renal cells as renal tissue sub-compartment.

The physiological parameters (subsections 4.2.2. and 4.2.3.) and experimentally observed metformin concentration time-courses (subsection 4.3.) were combined to build, parametrize and validate an ODE-based PBPK model. After that, validation of the model simulations at different parameter values was carried out.

#### 4.2.2. Parameters of mice model

The PBPK model of mice mathematically describes metformin pharmacokinetics in plasma and 11 organs, using 20 compartments (Fig 9). The model was parameterized using physiological parameters such as tissue size [58–60], blood flow rates [58, 61] and gut motility. The small intestine was parametrized based on the experimental data of human cell line Caco-2 data [35]. The estimation of unknown parameters was based on the experimental metformin concentration time-course data published by Wilcock and Bailey [1]. All the parameters of the mice model are compiled in the supplementary file **S1 Table**.

#### 4.2.3. Parameters of human model

The human model mathematically describes metformin pharmacokinetics in plasma, red blood cells, and 11 organs, using 21 compartments (Fig 9). Published physiological parameters of tissue size [58, 59, 62], blood flow rates [58, 63] and kinetic parameters of reactions [58, 61, 64, 65] were applied. K_t:p_ coefficients were taken from the mice model. The absorption and elimination coefficients were introduced to scale-up parameters of the small intestine and kidney correspondingly. All the parameters of the human model are compiled in the supplementary file **S2 Table**.

### 4.3. Experimental data sets

In total, seven metformin pharmacokinetic datasets by different authors were used to create and validate the mice and human models. The unknown parameters in the mice model were estimated using two datasets from Wilcock & Bailey, where metformin concentrations in various tissues were measured over a 24h period following 50 mg/kg IV and PO doses of metformin. The model was then adapted for humans, and the unknown parameters were estimated using two datasets–a dataset from Zaharenko following a 500mg PO dose [20] and a dataset from Chung following a 1000mg PO dose of metformin hydrochloride [36]. The two datasets at 500mg and 1000mg were used because they represent the most commonly prescribed metformin hydrochloride doses and to ensure that the estimated parameters are fit to a wider spectrum of dose administration. To test the accuracy of the developed model, the model predictions were validated against four independent datasets with different PO doses of metformin hydrochloride - 250mg dose dataset from Chung [36], 500mg dose dataset from Gusler [37], 750mg dose dataset from Wen [38] and a 500mg multiple dose dataset from El Messaoudi [39]. In the case of humans, metformin has been administered in tablets as metformin hydrochloride. Therefore the doses of metformin hydrochloride were recalculated to metformin doses according to the proportion of molecular mass to the free metformin base before using them in simulations (1000mg metformin hydrochloride corresponds to 778.4mg of metformin). The datasets used in PBPK model development are described in the following subsections.

#### 4.3.1. Mice datasets

The data in the Wilcock study were collected from two separate experiments following peroral and intravenous doses of 50mg/kg in mice. The Wilcock datasets after the PO administration contain metformin concentration time-course data from different tissues (stomach, small intestine, colon, liver, kidney, heart, skeletal muscle, white fat, brain, and submaxillary salivary gland) and plasma (taken from inferior vena cava (IVC) and hepatic portal vein (HPV)). The dataset for the IV dose contains metformin concentrations measured in plasma from IVC and the tissues of the stomach, small intestine, and liver. The data was collected at 0.5, 1, 2, 4, 8, and 24 h after the PO dose and 0.5, 1, 2, and 4 h after the IV dose. The published tissue concentrations were recalculated using the following equation, as Wilcock et al. had corrected the tissue concentrations by taking into account the inulin space in the respective organs [1]:

$$Tissue\;concentration=\frac{C}{FI}$$ (1)

where C—respective tissue concentration of metformin calculated by Wilcock et al., expressed as micromoles of metformin/kg of organ tissue and *FI—*an intracellular fraction of respective organ [66].

The experimental data also include metformin amount excreted in urine and feces since it is known from the Wilcock experimental data [1] that following the PO dose, 61% of the dose was excreted in the urine, and it is assumed that the remaining 39% are excreted in feces whereas following the IV dose all metformin was excreted in the urine.

Some data was excluded prom parameter estimation data files. Stomach measurements were excluded from the PO data file (see **S1 Data**) due to a much higher metformin concentration compared to the IV data suggesting that metformin in the stomach lumen influenced the measurements. The 2h data point in venous plasma, portal vein, and brain was excluded from the parameter estimation data file as it builds curves with two peaks that do not fit to the single peak character of metformin concentration changes. At the same time, those experimental data points are represented in Fig 1.

#### 4.3.2. Human datasets

Zaharenko dataset [20] was based on the mean values of nine healthy volunteers with available metformin concentration data in urine (**S3 Table**). The metformin concentrations for these volunteers were measured in plasma, red blood cells at time points 1, 2, 3, 4, 6, 10, 24 h after oral administration and in urine samples at time points 4, 6, 10, 24 h after oral administration of metformin.

Two datasets were used from Chung et al. in a study where ten healthy male volunteers were randomized to receive PO doses of 1000mg or 250mg of metformin hydrochloride. In the case of 1000mg dose administration, two doses of 1000mg were administered at 12-hour intervals, and the blood samples were collected at the start of the second dose administration at 0, 0.5, 1, 1.5, 2, 2.5, 3, 4, 6, 8, 10, and 12 hours [36]. In the case of 250mg dose administration, two doses of metformin hydrochloride were administered at 12-hour intervals, the first dose of 375mg and second of 250mg with the blood samples collected at the start of the second dose administration at 0, 0.5, 1, 1.5, 2, 2.5, 3, 4, 6, 8, 10, and 12 hours [36].

The dataset from Gusler et al. [37] contains metformin concentration data in plasma from 14 healthy individuals following a single peroral metformin hydrochloride dose of 500mg. The blood samples were taken at 0, 0.5, 1, 1.5, 2, 3, 4, 6, 8, 10, 12, 14, 16, 20, 24 hours after dose administration.

The dataset from Wen et al. [38] contains metformin concentration data in plasma from 8 healthy individuals following two peroral metformin hydrochloride doses of 500mg and 750mg at a 12-hour interval. The blood samples were taken 12 hours after the first 500mg dose at the start of the second 750mg dose administration at 0, 0.5, 1, 1.5, 2, 2.5, 3, 3.5, 4, 6, 8, 10, and 12 hours.

The dataset from El Messaoudi et al. [39] contains metformin concentration data in plasma from 17 healthy individuals following a four-day long twice-daily peroral administration of 500mg of metformin hydrochloride. The blood samples were taken at the start of the third day at 0, 1, 1.5, 2, 2.5, 3, 3.5, 4, 5, 6, 8, and 10 hours.

### 4.4. Physiological assumptions

#### 4.4.1. Absorption of metformin

When metformin is administrated orally, the gastrointestinal absorption from the immediate-release dosage form is incomplete—around 40% of an administered dose is excreted with feces [1, 42, 67]. Intestinal permeability demonstrated by metformin is concentration- and site-dependent [50]. It has been shown, that metformin is not appreciably absorbed from the stomach and the large intestine [67]. During the model development phase, it was assumed that the concentration measured in the stomach compartment is due to the blood flow to this particular compartment and that absorption processes do not have any influence on measured concentration in the stomach. The absorption of metformin is confined very largely to the small intestine [42, 50, 67], therefore in the model, the small intestine is the only organ where the absorption takes place. It has been described that the uptake of metformin relays on intestinal transporters involved in the absorptive transport of metformin, as metformin is a well-known substrate of the organic cation transporters (OCT) like OCT1-3 and the multidrug and toxin extrusion transporters (MATE) like MATE1 and MATE2-K [42, 68, 69]. Furthermore, as metformin is the hydrophilic and positively charged molecule at either gastrointestinal or intracellular pH, it is expected that transport proteins are required to facilitate its transcellular movement. However, this assumption has been challenged, and it is suggested that charged and uncharged hydrophilic compounds such as metformin with specific characteristics like exceptionally strong basicity and small molecular size, could be absorbed by a concentration gradient-driven passive diffusion and facilitated diffusion via paracellular transport [35]. Based on these assumptions, the small intestine compartment was developed as three independent compartments (Fig 2A) with active transport carried out by OCT and two types of diffusion transport). The values of uptake parameters from the gastrointestinal lumen (the maximal velocity (V_max_), the Michaelis-Menten constant (K_m,_), and the non-saturable component of uptake (K_d_)) established Caco-2 cell monolayers by Proctor were recalculated [35]. The original parameters were calculated per mg protein and were recalculated to account for the surface area–it was considered that the average small intestinal area is around 3m^2^ for mice and 70 m^2^ for humans and that 1 cm^2^ of monolayer contains 0.2 mg of protein. The amount of protein in the monolayer is also included as a parameter in the human model as “Proctor coefficient”.

In the developed PBPK model, the small intestine was considered as one unit that is described by the above-mentioned intestinal compartments (Fig 10A), and the average concentration from different intestinal regions was calculated from the published data by Wilcock et al. [1].

#### 4.4.2. Distribution

The tissue distribution equation incorporated within the PBPK model provides a prediction for the tissue:plasma partition coefficients for the organs employed as compartments in the model. K_t:p_ was used to describe the steady-state metformin concentration in tissues in relation to the free concentration in plasma [22]. The K_t:p_ coefficients obtained by parameter estimation described the passive and active transport in various tissues [27] and were compared with the calculated values based on the prediction method developed by Rodgers et al. [14], which depends on compound characteristics such as lipophilicity (log P), pKa, and the fraction of the free drug in plasma, as well as on the composition of water, fat, and proteins in each of the tissues making up the compartments. However, since metformin is known to be transported into the cells only by active transport and passive diffusion is negligible, the K_t:p_ only describes the active transport and electrostatic interactions of metformin and acidic phospholipids. [27].

#### 4.4.3. Elimination

Systemic clearance is the measure of the ability of the body to eliminate a drug, and it is expressed as a volume per unit of time and usually is expressed sum of the respective clearances of eliminating organs [70]. The rapid systemic clearance of unchanged metformin is solely accounted for a very extensive renal elimination by excretion into the urine, involving both glomerular filtration and active tubular secretion of this ionized hydrophilic pharmaceutical ingredient [10, 17]. Therefore, the elimination of metformin mainly occurs as a result of excretion by the kidney, which allows us to assume that metformin systemic clearance is equal or highly close to the renal clearance. The organic cation transporters and toxin extrusion transporters have a significant role in metformin renal secretion by facilitating the secretion rate above the physiological unbound glomerular filtration rate (GFR) [47]. It simplifies the modeling of elimination as metformin is removed from the systemic circulation only when it passes through the eliminating organ—kidney. In the model, it assumed that the renal clearance consists of two components:

$${CL}_{renal}=Q_{GFR}+{CL}_{renal\;active}$$ (2)

where QGFR—Glomerular filtration rate, CL active—the active transporter-mediated renal clearance.

The developed structure of kidneys is described in section 4.2.1., where the excreted metformin flow is assumed to be a content of pre-urine, which is transferred from the proximal tubule to the distal tubule regions and further eliminated by urine. As no additional reabsorption of metformin in the distal regions of the tubule is expected to occur, the distal tubule and the collecting duct were not incorporated as a part of the sub-compartment model of the kidney. It has been previously described that the organic cation transporters (OCT 1 and 2) and the multidrug and toxin extrusion transporters (MATE1 and MATE2-K) have a high impact on the renal clearance of metformin [48, 71, 72]. The OCT2 transporters are located on the basolateral side of renal tubular cells and transport metformin into the tubular tissues, while MATE1, MATE2-K, and OCT1 have been found in the apical membranes of proximal tubules cells [42, 48]. In the model, it was assumed that MATE1 reaction represents activities of MATE2-K and OCT1 transporter as well [65, 72]. Only unbound metformin molecules can pass through cellular membranes. Due to that, it must partition out of the red blood cells to become available for renal elimination. As the metformin does not bind with plasma protein, the dissociation from red blood cells exclusively limits the excretion of metformin from the blood [70].

### 4.5. Scaling the model to humans

The human PBPK model was scaled-up based on the developed metformin PBPK model of mice applying human physiological parameters, such as organ volume and blood flow rate from a healthy subject weighing 70 kg (weight is an adjustable parameter in the model). The model parameters are listed in the supplementary file **S2 Table**. The estimated K_t:p_ values in tissues of mice were assumed to be identical in humans and recalculation was not done as metformin does not bind to plasma proteins [14]. The kinetic parameters for absorption were scaled to the surface area of the human intestine of 71m^2^, and since absorption of metformin is varying in the different parts of the small intestine, a coefficient for the relative transport activity was introduced. This parameter was determined using parameter estimation. A similar parameter was introduced in the reactions responsible for metformin elimination since it is a process involving active transporters–a scaling coefficient was introduced to account for the transporter expression differences between the mice and the human model. For the absorption, the kinetic coefficients in five reactions corresponding to metformin absorption from the intestine lumen with intracellular or transcellular transport (03.2. IntestineLumen -> Enterocytes (PMAT OCT3) Vf, 03.3. Enterocytes -> IntestineVascular (OCT1) Vmax, 03.4. IntestineLumen -> IntestineVascular (Saturable), 03.6. IntestineLumen -> Enterocytes (Diffusion) Coefficient, 03.7. IntestineLumen -> IntestineVascular (Diffusion) Coefficient) were multiplied by the scaling coefficient. In the elimination compartments, the kinetic coefficients of the two reactions regarding active transport (13.4. KidneyPlasma -> KidneyTissue, 13.5. KidneyTissue -> KidneyTubular) were multiplied by the scaling coefficient.

## Supporting information

S1 Table List of mice model parameters and their description. (XLSX)

S2 Table List of human model parameters and their description. (XLSX)

S3 Table Metformin concentration time-series data for nine healthy individuals from Zaharenko et al., 2016. (XLSX)

S1 Text Description of models and their simulation using *COPASI*. (DOCX)

S1 Data Human and mice model files in SBML and COPASI formats, equations and experimental data files for parameter fitting and validation. (ZIP)
