# Supplementary material for: Physiologically based metformin pharmacokinetics model of mice and scale-up to humans for the estimation of concentrations in various tissues
Source: PLoS One. 2021 Apr 7;16(4):e0249594. doi: 10.1371/journal.pone.0249594 (PMC8026019; doi:10.1371/journal.pone.0249594)
Supplement: S1 Text — (DOCX) [file pone.0249594.s004.docx]

**S1 Text. Description of models and their simulation using *COPASI* (MS Word)**

Darta Maija Zake, Janis Kurlovics, Linda Zaharenko, Vitalijs Komasilovs, Janis Klovins and Egils Stalidzans (2021) Physiologically based metformin pharmacokinetics model of mice and scale-up to humans for the estimation of concentrations in various tissues, PLOS ONE, doi: 10.1371/journal.pone.0249594

**PBPK models of metformin in mice and humans**

Mice (single dose intravenous and single dose peroral) and human (single dose peroral and multiple dose peroral) models available in supplementary material **S1 Data** and can be simulated as they stand by opening the files with extension “.cps” with the freely available COPASI software ([www.copasi.org](http://www.copasi.org)) where the time courses of metformin concentrations and the amount in tissues as well as transport rates are pre-defined. SBML Level 2 Version 4 model files with extension “.xml” can be opened with SBML compatible software (see <http://sbml.org/SBML_Software_Guide>). The numerical values of model parameters along with their sources are described in the supplementary materials – **S1 Table for mice** model and **S2 Table for human** model. The structure of the model with reaction numbers is presented in Fig.1.

**Time course simulations in COPASI can be accessed through the section “Time Course”** in this section the time duration and intervals can be changed. When time-course simulations are run three plots are created – 1) metformin amount in compartments in mg, 2) metformin concentrations in the compartments (nmol/mL) and 3) reaction fluxes of all the reactions (nmol/h).

Look at “Output Specifications” -> “Plots” to activate or deactivate plots.

The time points of dose release are defined as “events” in COPASI and can be changed as necessary.

**Mice PBPK model**

This is a whole-body model representing the pharmacokinetics of metformin in mice. The model is built as a set of ODE that describes metformin concentration changes in time in the model compartments.

The model contains **20 compartments** (“Compartments” in COPASI model) describing various tissues or tissue sub-compartments and body fluids of metformin action (lumen, plasma, urine). The body weight and the weight of all compartments is expressed as a volume in mL and for the calculations it is assumed that 1mL = 1g. The volumes of most compartments are calculated as a fraction of the body weight/volume, and the fractions are determined from literature data (see supplementary material **S1 Table**), the volumes of the stomach lumen and intestine lumen are fixed and do not change depending on the body weight. Similarly, the volume of external urine and feces is set to 1mL, because those are “volumeless” compartments: they are used only for the calculation of metformin amount, not concentration.

The model contains **20 species** (“Species” in COPASI model) that correspond to the metformin concentrations in the 20 compartments. The initial concentrations for all the species is 0 nmol/mL as metformin is not produced in the body and can only be detected after dose administration.


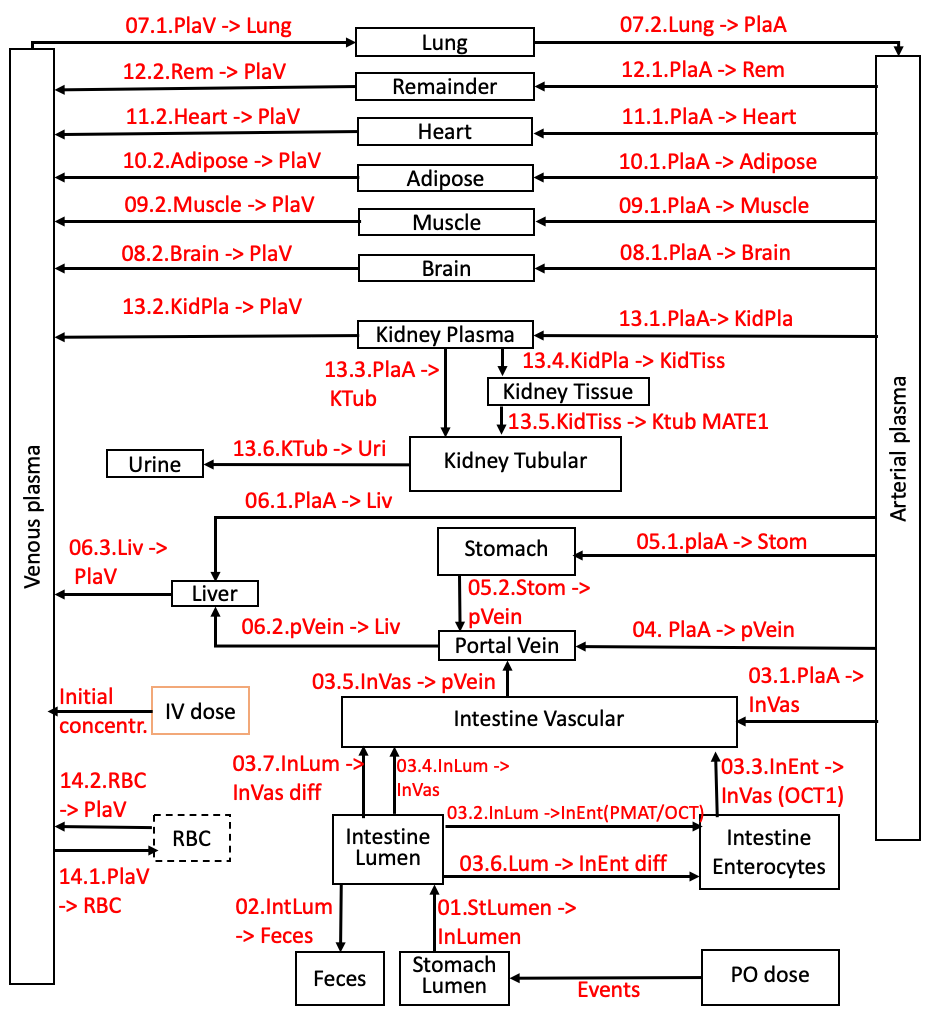


**Fig.1.** The structure of models and localization of reactions. The compartment RBC (red blood cells) and reactions 14.1. and 14.2. are applied only in the human model.

The model contains **33 reactions** (“Reactions” in COPASI model) – they describe the transport processes of metformin in the body. The reactions include ***local*** parameters that are involved only in that particular reaction and ***global parameters*** – parameters that are used in multiple reactions or are calculated depending on another parameter e.g. scale-up coefficients.

The model contains of **52 global quantities** – parameters involved in multiple reactions or necessary for another parameter calculation:

1. Parameters defining metformin dose – either peroral (global quantity “Metformin Dose in Lumen in mg”) where the dose is assigned to the stomach lumen or intravenous (global quantity “Metformin Dose in Plasma in mg”) where the dose is assigned to venous plasma.
2. Parameters describing the physiology – body weight (in mL), cardiac output, blood flow to different compartments described as Q”compartment_name” (for example “Qliver” describes blood flow to the liver compartment).”Qgfr” refers to the glomerular filtration rate.
3. Tissue specific tissue:plasma partition coefficients K_t:p_ (for example “Ktp_muscle” in the model).
4. Parameters involved in the calculation of metformin amount in mg in compartments, these parameters are named “mgCompartment_name” (for example “mgLiver” describes the metformin amount in mg in the liver tissues).

The time points of dose release are defined as “events” in COPASI and can be changed as necessary.

Time course simulations can be accessed through the section “Time Course” in this section the time duration and intervals can be changed. When the time-course simulations are run three plots are created – 1) metformin amount in the 20 compartments, 2) metformin concentrations in the compartments and 3) reaction fluxes of all the reactions (see “Output Specifications” -> “Plots” to activate or deactivate plots).

**Human PBPK model**

The PBPK model for humans is a whole-body model extrapolated from the PBPK model of mice taking into account that many physiological processes have similar mechanisms.

Following changes have been made during extrapolation of mice model to human model:

1. Physiological parameters of mice have been replaced by the human ones: 1) whole body mass and the distribution of weight over tissues and 2) cardiac output and distribution of blood flow over tissues.
2. An extra compartment for red blood cells is introduced (**21 compartments in total**) and a concentration of metformin in red blood cells is introduced (**21 species in total**) because of the availability of red blood cell experimental data for humans.
3. Two reactions are added (**35 reactions in total**) to represent the in-flux and out-flux of metformin in red blood cells.
4. The number of global quantities is increased (62 global quantities in total) to implement scale-up from the mice to the human model.
5. Parameters involved in the scale-up of the model (**62 global quantities**)
   1. “Kidney Coefficient” is used for the scale-up coefficient of metformin elimination and is involved in the calculation of the reaction rate parameters in the reactions ﻿“13.4. KidneyPlasma -> KidneyTissue” and “﻿13.5. KidneyTissue -> KidneyTubular”. This parameter was determined using parameter estimation.
   2. “IntestineSurfaceCoefficient” (= Human intestine surface/mouse intestine surface=70/0.032=2187.5) is used to scale-up the intestine surface from mice to human.
   3. “Intestine Coefficient” is used for the scale-up coefficient of metformin absorption intestinal reaction rates of the reactions (“03.2. IntestineLumen -> Enterocytes (PMAT OCT3)”, ﻿ “03.3. Enterocytes -> IntestineVascular (OCT1)”, “03.4. IntestineLumen -> IntestineVascular (Saturable)”, “03.6. IntestineLumen -> Enterocytes (Diffusion)” and “03.7. IntestineLumen -> IntestineVascular (Diffusion)”). This parameter was determined using parameter estimation.

The time points of dose release are defined as “events” in COPASI and can be changed as necessary.

**Time course simulations can be accessed through the section “Time Course”** in this section the time duration and intervals can be changed. When time-course simulations are run three plots are created – Metformin amount in the 21 compartments, metformin concentrations in the compartments and reaction fluxes of all the reactions (see “Output Specifications” -> “Plots” to activate or deactivate plots).
