# Supplementary material for: Physiologically based metformin pharmacokinetics model of mice and scale-up to humans for the estimation of concentrations in various tissues
Source: PLoS One. 2021 Apr 7;16(4):e0249594. doi: 10.1371/journal.pone.0249594 (PMC8026019; doi:10.1371/journal.pone.0249594)
Supplement: S1 Data — (ZIP) [file pone.0249594.s005.zip › S1DataModelsAndExpData/Humans models/Human equations.pdf]

$$V_{Liver} = \text{"Values[Body Weight].InitialValue"} \cdot 0.0257$$
$$V_{KidneyPlasma} = \text{"Values[Body Weight].InitialValue"} \cdot 0.00146$$
$$V_{Remainder} = \text{"Values[Body Weight].InitialValue"} \cdot 0.233$$
$$V_{PlasmaVenous} = \text{"Values[Body Weight].InitialValue"} \cdot 0.0321$$
$$V_{Heart} = \text{"Values[Body Weight].InitialValue"} \cdot 0.005$$
$$V_{Muscle} = \text{"Values[Body Weight].InitialValue"} \cdot 0.4$$
$$V_{Brain} = \text{"Values[Body Weight].InitialValue"} \cdot 0.02$$
$$V_{Adipose} = \text{"Values[Body Weight].InitialValue"} \cdot 0.215$$
$$V_{PlasmaArterial} = \text{"Values[Body Weight].InitialValue"} \cdot 0.0107$$
$$V_{Lung} = \text{"Values[Body Weight].InitialValue"} \cdot 0.0076$$
$$V_{PortalVein} = \text{"Values[Body Weight].InitialValue"} \cdot 0.0028$$
$$V_{Stomach} = \text{"Values[Body Weight].InitialValue"} \cdot 0.0021$$
$$V_{Enterocytes} = \text{"Values[Body Weight].InitialValue"} \cdot 0.0012$$
$$V_{KidneyTissue} = \text{"Values[Body Weight].InitialValue"} \cdot 0.00146$$
$$V_{IntestineVascular} = \text{"Values[Body Weight].InitialValue"} \cdot 0.009$$
$$V_{RBC} = \text{"Values[Body Weight].InitialValue"} \cdot 0.0314$$
$$V_{KidneyTubular} = \text{"Values[Body Weight].InitialValue"} \cdot 0.00146$$

$$\begin{aligned}\frac{d([mLiver] \cdot V_{Liver})}{dt} = & -\left(\frac{\text{QLiverOut} \cdot \text{[mLiver]}}{\text{Ktp\_Liver}}\right) \\ & +(\text{QPortalVeinOut} \cdot \text{[mPortalVein]}) \\ & +(\text{QLiverArtery} \cdot \text{[mPlasmaArterial]})\end{aligned}$$

$$\begin{aligned}\frac{d([mKidneyPlasma] \cdot V_{KidneyPlasma})}{dt} = & -\left(\frac{\text{"13.4. KidneyPlasma -> KidneyTissue"} \cdot \text{[mKidneyPlasma]}}{Km_{(\text{"13.4. KidneyPlasma -> KidneyTissue"})} + \text{[mKidneyPlasma]}}\right) \\ & -\left(\frac{\text{QKidney} \cdot \text{[mKidneyPlasma]}}{\text{Ktp\_Kidney}}\right) \\ & +(\text{QKidney} \cdot \text{[mPlasmaArterial]}) \\ & -(\text{"mArterialPlasma (kidney)"} \cdot \text{Qgfr})\end{aligned}$$

$$\begin{aligned}\frac{d([mRemainder] \cdot V_{Remainder})}{dt} = & -\left(\frac{\text{QRemainder} \cdot \text{[mRemainder]}}{\text{Ktp\_Remainder}}\right) \\ & +(\text{QRemainder} \cdot \text{[mPlasmaArterial]})\end{aligned}$$

$$\begin{aligned}\frac{d([mPlasmaVenous] \cdot V_{PlasmaVenous})}{dt} = & +\left(\frac{\text{QLiverOut} \cdot \text{[mLiver]}}{\text{Ktp\_Liver}}\right) \\ & +\left(\frac{\text{QKidney} \cdot \text{[mKidneyPlasma]}}{\text{Ktp\_Kidney}}\right) \\ & -(\text{"Cardiac Output"} \cdot \text{[mPlasmaVenous]}) \\ & +\left(\frac{\text{QBrain} \cdot \text{[mBrain]}}{\text{Ktp\_Brain}}\right) \\ & +\left(\frac{\text{QHeart} \cdot \text{[mHeart]}}{\text{Ktp\_Heart}}\right) \\ & +\left(\frac{\text{QAdipose} \cdot \text{[mAdipose]}}{\text{Ktp\_Adipose}}\right) \\ & +\left(\frac{\text{QMuscle} \cdot \text{[mMuscle]}}{\text{Ktp\_Muscle}}\right) \\ & -(k1_{(\text{"14.1. PlasmaVenous -> RBC"})} \cdot \text{[mPlasmaVenous]}) \\ & +(k1_{(\text{"14.2. RBC -> PlasmaVenous"})} \cdot \text{[mRBC]}) \\ & +\left(\frac{\text{QRemainder} \cdot \text{[mRemainder]}}{\text{Ktp\_Remainder}}\right)\end{aligned}$$

$$\begin{aligned}\frac{d([mHeart] \cdot V_{Heart})}{dt} = & -\left(\frac{\text{QHeart} \cdot \text{[mHeart]}}{\text{Ktp\_Heart}}\right) \\ & +(\text{QHeart} \cdot \text{[mPlasmaArterial]})\end{aligned}$$

$$\begin{aligned}\frac{d([mMuscle] \cdot V_{Muscle})}{dt} = & -\left(\frac{\text{QMuscle} \cdot \text{[mMuscle]}}{\text{Ktp\_Muscle}}\right) \\ & +(\text{QMuscle} \cdot \text{[mPlasmaArterial]})\end{aligned}$$

$$\begin{aligned}\frac{d([mAdipose] \cdot V_{Adipose})}{dt} = & -\left(\frac{\text{QAdipose} \cdot \text{[mAdipose]}}{\text{Ktp\_Adipose}}\right) \\ & +(\text{QAdipose} \cdot \text{[mPlasmaArterial]})\end{aligned}$$

$$\begin{aligned}\frac{d([mBrain] \cdot V_{Brain})}{dt} = & -\left(\frac{\text{QBrain} \cdot \text{[mBrain]}}{\text{Ktp\_Brain}}\right) \\ & +(\text{QBrain} \cdot \text{[mPlasmaArterial]})\end{aligned}$$

$$\frac{d([mFeces] \cdot V_{Feces})}{dt} = +(k1_{(\text{"02. IntestineLumen -> Feces"})} \cdot \text{[mIntestineLumen]})$$

$$\frac{d([mUrineExternal] \cdot V_{Urine})}{dt} = +(k1_{(\text{"13.6. KidneyTubular -> UrineExternal"})} \cdot \text{[mKidneyTubular]})$$

$$\begin{aligned}\frac{d([mIntestineLumen] \cdot V_{IntestineLumen})}{dt} = & -\left(\frac{\text{"03.2. IntestineLumen -> Enterocytes (PMAT OCT3) Vf"} \cdot \text{[mIntestineLumen]}}{Km_{(\text{"03.2. IntestineLumen -> Enterocytes (PMAT OCT3)"})} + \text{[mIntestineLumen]}}\right) \\ & -\left(\frac{\text{"03.4. IntestineLumen -> IntestineVascular (Saturable)"} \cdot \text{[mIntestineLumen]}}{Km_{(\text{"03.4. IntestineLumen -> IntestineVascular (Saturable)"})} + \text{[mIntestineLumen]}}\right) \\ & +(k1_{(\text{"01. StomachLumen -> IntestineLumen"})} \cdot \text{[mStomachLumen]}) \\ & -((\text{"03.6. IntestineLumen -> Enterocytes (Diffusion) Coefficient"} \cdot \text{[mIntestineLumen]} - \text{"03.6. IntestineLumen -> Enterocytes (Diffusion) Coefficient"} \cdot \text{[mEnterocytes]})) \\ & -((\text{"03.7. IntestineLumen -> IntestineVascular (Diffusion) Coefficient"} \cdot \text{[mIntestineLumen]} - \text{"03.7. IntestineLumen -> IntestineVascular (Diffusion) Coefficient"} \cdot \text{[mIntestineVascular]})) \\ & -(k1_{(\text{"02. IntestineLumen -> Feces"})} \cdot \text{[mIntestineLumen]})\end{aligned}$$

$$\begin{aligned}\frac{d([mPlasmaArterial] \cdot V_{PlasmaArterial})}{dt} = & -(\text{QStomach} \cdot \text{[mPlasmaArterial]}) \\ & -(\text{QPortalVein} \cdot \text{[mPlasmaArterial]}) \\ & -(\text{QIntestineVascular} \cdot \text{[mPlasmaArterial]}) \\ & -(\text{QLiverArtery} \cdot \text{[mPlasmaArterial]}) \\ & +\left(\frac{\text{"Cardiac Output"} \cdot \text{[mLung]}}{\text{Ktp\_Lung}}\right) \\ & -(\text{QBrain} \cdot \text{[mPlasmaArterial]}) \\ & -(\text{QHeart} \cdot \text{[mPlasmaArterial]}) \\ & -(\text{QAdipose} \cdot \text{[mPlasmaArterial]}) \\ & -(\text{QKidney} \cdot \text{[mPlasmaArterial]}) \\ & -(\text{QMuscle} \cdot \text{[mPlasmaArterial]}) \\ & -(\text{QRemainder} \cdot \text{[mPlasmaArterial]})\end{aligned}$$

$$\begin{aligned}\frac{d([mLung] \cdot V_{Lung})}{dt} = & -\left(\frac{\text{"Cardiac Output"} \cdot \text{[mLung]}}{\text{Ktp\_Lung}}\right) \\ & +(\text{"Cardiac Output"} \cdot \text{[mPlasmaVenous]})\end{aligned}$$

$$\begin{aligned}\frac{d([mPortalVein] \cdot V_{PortalVein})}{dt} = & +\left(\frac{\text{QIntestineVascular} \cdot \text{[mIntestineVascular]}}{\text{Ktp\_IntestineVascular}}\right) \\ & -(\text{QPortalVeinOut} \cdot \text{[mPortalVein]}) \\ & +\left(\frac{\text{QStomach} \cdot \text{[mStomach]}}{\text{Ktp\_Stomach}}\right) \\ & +(\text{QPortalVein} \cdot \text{[mPlasmaArterial]})\end{aligned}$$

$$\begin{aligned}\frac{d([mStomach] \cdot V_{Stomach})}{dt} = & +(\text{QStomach} \cdot \text{[mPlasmaArterial]}) \\ & -\left(\frac{\text{QStomach} \cdot \text{[mStomach]}}{\text{Ktp\_Stomach}}\right)\end{aligned}$$

$$\begin{aligned}\frac{d([mEnterocytes] \cdot V_{Enterocytes})}{dt} = & +\left(\frac{\text{"03.2. IntestineLumen -> Enterocytes (PMAT OCT3) Vf"} \cdot \text{[mIntestineLumen]}}{Km_{(\text{"03.2. IntestineLumen -> Enterocytes (PMAT OCT3)"})} + \text{[mIntestineLumen]}}\right) \\ & -\left(\frac{\text{"03.3. Enterocytes -> IntestineVascular (OCT1) Vmax"} \cdot \text{[mEnterocytes]}}{Km_{(\text{"03.3. Enterocytes -> IntestineVascular (OCT1)"})} + \text{[mEnterocytes]}}\right) \\ & +((\text{"03.6. IntestineLumen -> Enterocytes (Diffusion) Coefficient"} \cdot \text{[mIntestineLumen]} - \text{"03.6. IntestineLumen -> Enterocytes (Diffusion) Coefficient"} \cdot \text{[mEnterocytes]}))\end{aligned}$$

$$\begin{aligned}\frac{d([mKidneyTissue] \cdot V_{KidneyTissue})}{dt} = & +\left(\frac{\text{"13.4. KidneyPlasma -> KidneyTissue"} \cdot \text{[mKidneyPlasma]}}{Km_{(\text{"13.4. KidneyPlasma -> KidneyTissue"})} + \text{[mKidneyPlasma]}}\right) \\ & -\left(\frac{\text{"13.5. KidneyTissue -> KidneyTubular"} \cdot \text{[mKidneyTissue]}}{Km_{(\text{"13.5. KidneyTissue -> KidneyTubular"})} + \text{[mKidneyTissue]}}\right)\end{aligned}$$

$$\begin{aligned}\frac{d([mIntestineVascular] \cdot V_{IntestineVascular})}{dt} = & -\left(\frac{\text{QIntestineVascular} \cdot \text{[mIntestineVascular]}}{\text{Ktp\_IntestineVascular}}\right) \\ & +(\text{QIntestineVascular} \cdot \text{[mPlasmaArterial]}) \\ & +\left(\frac{\text{"03.4. IntestineLumen -> IntestineVascular (Saturable)"} \cdot \text{[mIntestineLumen]}}{Km_{(\text{"03.4. IntestineLumen -> IntestineVascular (Saturable)"})} + \text{[mIntestineLumen]}}\right) \\ & +\left(\frac{\text{"03.3. Enterocytes -> IntestineVascular (OCT1) Vmax"} \cdot \text{[mEnterocytes]}}{Km_{(\text{"03.3. Enterocytes -> IntestineVascular (OCT1)"})} + \text{[mEnterocytes]}}\right) \\ & +((\text{"03.7. IntestineLumen -> IntestineVascular (Diffusion) Coefficient"} \cdot \text{[mIntestineLumen]} - \text{"03.7. IntestineLumen -> IntestineVascular (Diffusion) Coefficient"} \cdot \text{[mIntestineVascular]}))\end{aligned}$$

$$\frac{d([mStomachLumen] \cdot V_{StomachLumen})}{dt} = -(k1_{(\text{"01. StomachLumen -> IntestineLumen"})} \cdot \text{[mStomachLumen]})$$

$$\begin{aligned}\frac{d([mRBC] \cdot V_{RBC})}{dt} = & +(k1_{(\text{"14.1. PlasmaVenous -> RBC"})} \cdot \text{[mPlasmaVenous]}) \\ & -(k1_{(\text{"14.2. RBC -> PlasmaVenous"})} \cdot \text{[mRBC]})\end{aligned}$$

$$\begin{aligned}\frac{d([mKidneyTubular] \cdot V_{KidneyTubular})}{dt} = & +\left(\frac{\text{"13.5. KidneyTissue -> KidneyTubular"} \cdot \text{[mKidneyTissue]}}{Km_{(\text{"13.5. KidneyTissue -> KidneyTubular"})} + \text{[mKidneyTissue]}}\right) \\ & -(k1_{(\text{"13.6. KidneyTubular -> UrineExternal"})} \cdot \text{[mKidneyTubular]}) \\ & +(\text{"mArterialPlasma (kidney)"} \cdot \text{Qgfr})\end{aligned}$$

$$\text{QAdipose} = \text{"Values[Cardiac Output].InitialValue"} \cdot 0.052$$
$$\text{QBrain} = \text{"Values[Cardiac Output].InitialValue"} \cdot 0.114$$
$$\text{QHeart} = \text{"Values[Cardiac Output].InitialValue"} \cdot 0.04$$
$$\text{QKidney} = \text{"Values[Cardiac Output].InitialValue"} \cdot 0.175$$
$$\text{QMuscle} = \text{"Values[Cardiac Output].InitialValue"} \cdot 0.191$$
$$\text{QRemainder} = \text{"Values[Cardiac Output].InitialValue"} \cdot 0.201$$
$$\text{mgLiver} = \frac{129.16 \cdot \text{[mLiver]} \cdot \text{Compartments[Liver].InitialVolume}}{1000000}$$
$$\text{mgIntestineLumen} = \frac{129.16 \cdot \text{[mIntestineLumen]} \cdot \text{Compartments[IntestineLumen].InitialVolume}}{1000000}$$
$$\text{mgBrain} = \frac{129.16 \cdot \text{[mBrain]} \cdot \text{Compartments[Brain].InitialVolume}}{1000000}$$
$$\text{mgAdipose} = \frac{129.16 \cdot \text{[mAdipose]} \cdot \text{Compartments[Adipose].InitialVolume}}{1000000}$$
$$\text{mgHeart} = \frac{129.16 \cdot \text{[mHeart]} \cdot \text{Compartments[Heart].InitialVolume}}{1000000}$$
$$\text{mgKidneyPlasma} = \frac{129.16 \cdot \text{[mKidneyPlasma]} \cdot \text{Compartments[KidneyPlasma].InitialVolume}}{1000000}$$
$$\text{mgRemainder} = \frac{129.16 \cdot \text{[mRemainder]} \cdot \text{Compartments[Remainder].InitialVolume}}{1000000}$$
$$\text{mgMuscle} = \frac{129.16 \cdot \text{[mMuscle]} \cdot \text{Compartments[Muscle].InitialVolume}}{1000000}$$
$$\text{mgPortalVein} = \frac{129.16 \cdot \text{[mPortalVein]} \cdot \text{Compartments[PortalVein].InitialVolume}}{1000000}$$
$$\text{mgUrineSum} = \frac{129.16 \cdot \text{[mUrineExternal]} \cdot \text{Compartments[Urine].InitialVolume}}{1000000}$$
$$\text{mgFeces} = \frac{129.16 \cdot \text{[mFeces]} \cdot \text{Compartments[Feces].InitialVolume}}{1000000}$$
$$\text{mgEnterocytes} = \frac{129.16 \cdot \text{[mEnterocytes]} \cdot \text{Compartments[Enterocytes].InitialVolume}}{1000000}$$
$$\text{mgVenousPlasma} = \frac{129.16 \cdot \text{[mPlasmaVenous]} \cdot \text{Compartments[PlasmaVenous].InitialVolume}}{1000000}$$
$$\text{mgArterialPlasma} = \frac{129.16 \cdot \text{[mPlasmaArterial]} \cdot \text{Compartments[PlasmaArterial].InitialVolume}}{1000000}$$
$$\text{mgLung} = \frac{129.16 \cdot \text{[mLung]} \cdot \text{Compartments[Lung].InitialVolume}}{1000000}$$
$$\text{mgStomach} = \frac{129.16 \cdot \text{[mStomach]} \cdot \text{Compartments[Stomach].InitialVolume}}{1000000}$$
$$\text{QLiverArtery} = \text{"Values[Cardiac Output].InitialValue"} \cdot 0.046$$
$$\text{QStomach} = \text{"Values[Cardiac Output].InitialValue"} \cdot 0.011$$
$$\text{QPortalVein} = \text{"Values[Cardiac Output].InitialValue"} \cdot 0.064$$
$$\text{QIntestineVascular} = \text{"Values[Cardiac Output].InitialValue"} \cdot 0.106$$
$$\text{QLiverOut} = \text{Values[QPortalVeinOut].InitialValue} + \text{Values[QLiverArtery].InitialValue}$$
$$\text{mIntestineTotal} = \frac{\text{[mEnterocytes]} \cdot \text{Compartments[Enterocytes].InitialVolume} + \text{Compartments[IntestineVascular].InitialVolume} \cdot \text{[mIntestineVascular]}}{\text{Compartments[Enterocytes].InitialVolume} + \text{Compartments[IntestineVascular].InitialVolume}}$$
$$\text{mKidneyTotal} = \frac{\text{Compartments[KidneyPlasma].InitialVolume} \cdot \text{[mKidneyPlasma]} + \text{Compartments[KidneyTissue].InitialVolume} \cdot \text{[mKidneyTissue]} + \text{Compartments[KidneyTubular].InitialVolume} \cdot \text{[mKidneyTubular}\ldots}{\text{Compartments[KidneyPlasma].InitialVolume} + \text{Compartments[KidneyTissue].InitialVolume} + \text{Compartments[KidneyTubular].InitialVolume}}$$ [illegible]
$$\text{mgKidneyTissues} = \frac{129.16 \cdot \text{Compartments[KidneyTissue].InitialVolume} \cdot \text{[mKidneyTissue]}}{1000000}$$
$$\text{mgIntestineVascular} = \frac{129.16 \cdot \text{[mIntestineVascular]} \cdot \text{Compartments[IntestineVascular].InitialVolume}}{1000000}$$
$$\text{mgStomachLumen} = \frac{129.16 \cdot \text{[mStomachLumen]} \cdot \text{Compartments[StomachLumen].InitialVolume}}{1000000}$$
$$\text{QPortalVeinOut} = \text{Values[QStomach].InitialValue} + \text{Values[QPortalVein].InitialValue} + \text{Values[QIntestineVascular].InitialValue}$$
$$\text{"mArterialPlasma (kidney)"} = \text{[mPlasmaArterial]}$$
$$\text{mgKidneyTubular} = \frac{129.16 \cdot \text{Compartments[KidneyTubular].InitialVolume} \cdot \text{[mKidneyTubular]}}{1000000}$$
$$\text{mgRBC} = \frac{129.16 \cdot \text{[mRBC]} \cdot \text{Compartments[RBC].InitialVolume}}{1000000}$$
$$\text{"03.2. IntestineLumen -> Enterocytes (PMAT OCT3) Vf"} = \text{"Values[Intestine Coefficient].InitialValue"} \cdot \text{Values[IntestineSurffaceCoefficient].InitialValue} \cdot 1271$$
$$\text{"03.3. Enterocytes -> IntestineVascular (OCT1) Vmax"} = \text{"Values[Intestine Coefficient].InitialValue"} \cdot \text{Values[IntestineSurffaceCoefficient].InitialValue} \cdot 495$$
$$\text{"03.4. IntestineLumen -> IntestineVascular (Saturable)"} = \text{"Values[Intestine Coefficient].InitialValue"} \cdot \text{Values[IntestineSurffaceCoefficient].InitialValue} \cdot 19.2$$
$$\text{"03.6. IntestineLumen -> Enterocytes (Diffusion) Coefficient"} = \text{"Values[Intestine Coefficient].InitialValue"} \cdot \text{Values[IntestineSurffaceCoefficient].InitialValue} \cdot 0.14$$
$$\text{"03.7. IntestineLumen -> IntestineVascular (Diffusion) Coefficient"} = \text{"Values[Intestine Coefficient].InitialValue"} \cdot \text{Values[IntestineSurffaceCoefficient].InitialValue} \cdot 0.26$$
$$\text{"13.4. KidneyPlasma -> KidneyTissue"} = \text{"Values[Kidney Coefficient].InitialValue"} \cdot 87339$$
$$\text{"13.5. KidneyTissue -> KidneyTubular"} = \text{"Values[Kidney Coefficient].InitialValue"} \cdot 3000$$
$$\text{mgKidneyTotal} = \text{mgKidneyTubular} + \text{mgKidneyPlasma} + \text{mgKidneyTissues}$$
$$\text{mgIntestineTotal} = \text{mgIntestineVascular} + \text{mgEnterocytes}$$
$$\text{IntestineSurffaceCoefficient} = \frac{70}{0.032}$$
