# Supplementary figures and images for: Physiologically based metformin pharmacokinetics model of mice and scale-up to humans for the estimation of concentrations in various tissues
Source: PLoS One. 2021 Apr 7;16(4):e0249594. doi: 10.1371/journal.pone.0249594 (PMC8026019; doi:10.1371/journal.pone.0249594)

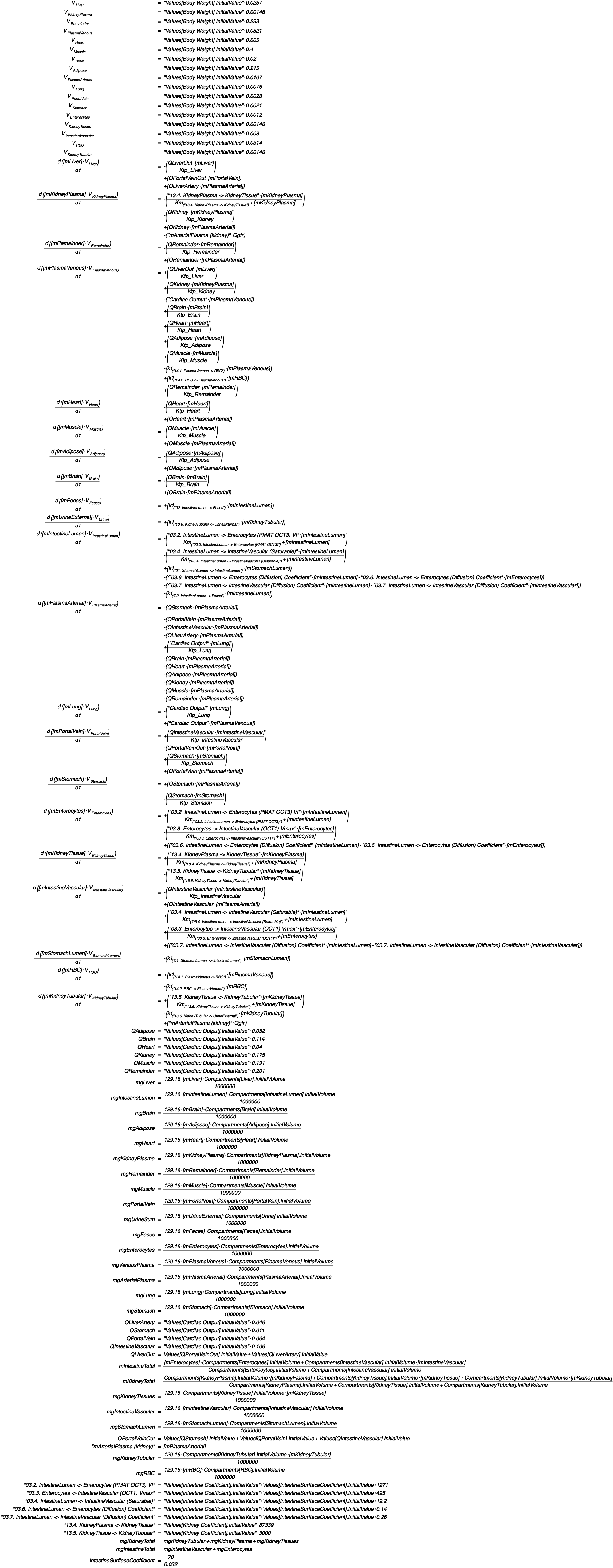

Supplement: S1 Data — (ZIP) [file pone.0249594.s005.zip › S1DataModelsAndExpData/Humans models/Human equations.png]

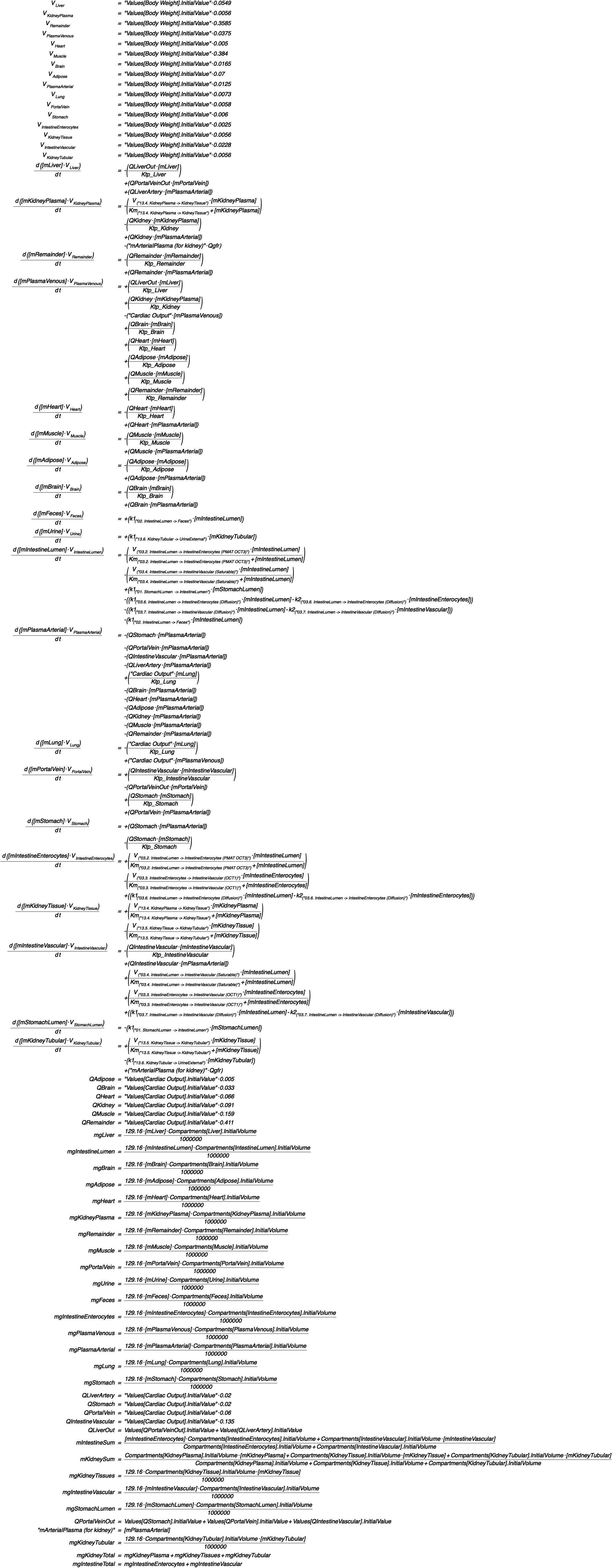

Supplement: S1 Data — (ZIP) [file pone.0249594.s005.zip › S1DataModelsAndExpData/Mice models/Mice equations.png]
